# Comprehensive transcriptome data to identify downstream genes of testosterone signalling in dermal papilla cells

**DOI:** 10.1038/s41597-022-01846-w

**Published:** 2022-11-29

**Authors:** Himari Matsusaka, Tao Wu, Kai Furuya, Tomoe Yamada-Kato, Lanlan Bai, Hiroshi Tomita, Eriko Sugano, Taku Ozaki, Tohru Kiyono, Isao Okunishi, Tomokazu Fukuda

**Affiliations:** 1 Graduate School of Science and Engineering, Iwate University, 4-3-5, Ueda, Morioka, Iwate 020-8551 Japan; 2 Research & Development Division, Kinjirushi corporation limited, 2-61 Yahata-hontori, Nakagawa-ku, Nagoya-shi, Aichi Japan; 3 Exploratory Oncology Research and Clinical Trial Center, National Cancer Center, 6-5-1 Kashiwanoha, Kashiwa, Chiba, 277–8577 Japan

**Keywords:** Endocrine cancer, Hormone receptors, Transcriptomics

## Abstract

Testosterone-related steroid hormones are associated with various types of diseases, including prostate cancer and androgenetic alopecia (AGA). The testosterone or dihydroxy testosterone (DHT) circulates through the blood, binds to the androgen receptor (AR) in the cytoplasm, and finally enters the nucleus to activate downstream target genes. We previously found that immortalized dermal papilla cells (DPCs) lost AR expression, which may be explained by the repeated cell passages of DPCs. To compensate for the AR expression, DPCs that express AR exogenously were established. In this study, we performed an RNA-Seq analysis of the AR-expressing and non-AR-expressing DPCs in the presence or absence of DHT to identify the downstream target genes regulated by AR signalling. Furthermore, we treated DPCs with minoxidil sulphate, which has the potential to treat AGA. This is the first comprehensive analysis to identify the downstream genes involved in testosterone signalling in DPCs. Our manuscript provides high-priority data for the discovery of molecular targets for prostate cancer and AGA.

## Abstract (summary)

**Table:** 

Measurement(s)	androgen receptor signaling pathway
Technology Type(s)	Next Generation Sequencing
Sample Characteristic - Organism	Homo

## Background & Summary

The metabolic form of testosterone, dihydroxy testosterone (DHT) has greater androgenic activity than the parental form of androgen^1^. This metabolic process is predominantly mediated by 5-alpha-reductase (5α-R)^2^. DHT has a much stronger affinity for the androgen receptor (AR) than the precursor form of testosterone. Due to the high affinity of DHT, understanding the pathogenesis of prostate cancer or androgenetic alopecia (AGA) may be important. However, the gene networks controlled by testosterone, especially in dermal papilla cells (DPCs) are not fully understood. Interestingly, we accidentally found that the expression of AR is strongly suppressed even in the early passage of human DPCs^3^. The suppression of AR gene expression under the cultured cell conditions of DPCs has been previously reported in rat-derived DPCs, which is consistent with our finding^4^. Therefore, our established immortalized DPCs are almost completely negative for AR expression. These situations led us to build the hypothesis that if we exogenously introduce AR via retrovirus, re-constitution of the AR signalling pathway might be possible in immortalized DPCs. We previously carried out the comparison between AR positive or negative DPCs with RNA-Seq, and identified that expression of caveolin-1 is suppressed in AR-expressing DPCs^5^. However, the downregulated caveolin-1 recovered after the DHT treatment, and this response was observed regardless of whether AR is expressed or not. Based on our previous experience trying to identify the downstream genes of AR, in this study, we set up the experimental design again to cover the gene expression change after DHT stimulation. We set up the AR negative and positive cells of DPCs, and no treated and treated cells with DHT. Furthermore, we classified AR downstream genes which are commonly upregulated or downregulated in AR-expressing cells and DHT-treated cells with AR expression. We then narrowed down the AR downstream genes that were not affected after DHT treatment under the absence of AR expression. Our high quality data would help scientists study the effect of AR signaling.

## Methods

### Cell lines

We obtained human primary DPCs from Promocell (Promocell, Heidelberg, Germany) through the local distributor, Takara Bio (Shiga, Japan). The primary cells were maintained in a Follicle Dermal Papilla Cell Growth Medium (Promocell) as per the manufacturer’s instructions. Cells immortalized by the expression of R24C-mutant CDK4, cyclin D1, and TERT have been described in our previous paper^3^. Based on the characteristics of the introduced genes (CDK4, cyclin D1, and TERT), we named this immortalized cell line HFDPC_K4DT. Furthermore, we introduced AR-expressing retrovirus into immortalized DPCs as described in our previous papers^3,5^.

### Experimental design and details of sequencing data

We set up 15 experimental groups to cover all aspects of the biological response to AR signalling activation. Each experimental group contains triplicate samples to ensure reproducibility.

Sample 1, HFDPC_K4DT_rep1, K4DT, intact immortalized DPCs.

Sample 2, HFDPC_K4DT_rep2, K4DT, intact immortalized DPCs.

Sample 3, HFDPC_K4DT_rep3, K4DT, intact immortalized DPCs.

Sample 4, HFDPC_K4DT_DHT_rep1, DHT, immortalized DPCs treated with 50 nM dihydroxy testosterone (DHT). No AR expression.

Sample 5, HFDPC_K4DT_DHT_rep2, DHT, immortalized DPCs treated with 50 nM dihydroxy testosterone (DHT). No AR expression.

Sample 6, HFDPC_K4DT_DHT_rep3, DHT, immortalized DPCs treated with 50 nM dihydroxy testosterone (DHT). No AR expression.

Sample 7, HFDPC_K4DT_AR_rep1, AR, immortalized DPCs with retroviral AR expression, No ligand treatment.

Sample 8, HFDPC_K4DT_AR_rep2, AR, immortalized DPCs with retroviral AR expression, No ligand treatment.

Sample 9, HFDPC_K4DT_AR_rep3, AR, immortalized DPCs with retroviral AR expression, No ligand treatment.

Sample 10, HFDPC_K4DT_AR_DHT_rep1, ARDHT, immortalized DPCs with retroviral AR expression, treated with 50 nM DHT.

Sample 11, HFDPC_K4DT_AR_DHT_rep2, ARDHT, immortalized DPCs with retroviral AR expression, treated with 50 nM of DHT.

Sample 12, HFDPC_K4DT_AR_DHT_rep3, ARDHT, immortalized DPCs with retroviral AR expression, treated with 50 nM of DHT.

Sample 13, HFDPC_K4DT_AR_DHT_mino_rep1, mino, immortalized DPCs with retroviral AR expression, treated with 50 nM of DHT and 30 nM of Minoxidil sulphate (MXS)

Sample 14, HFDPC_K4DT_AR_DHT_mino_rep2, mino, immortalized DPCs with retroviral AR expression, treated with 50 nM of DHT and 30 nM of MXS

Sample 15, HFDPC_K4DT_AR_DHT_mino_rep3, mino, immortalized DPCs with retroviral AR expression, treated with 50 nM of DHT and 30 nM of MXS

### Cell treatment and RNA extraction

The cells were treated with DHT or MXS for 24 h at approximately 70% confluence in 35 mm diameter cell culture dish. The total cellular RNA was extracted using the Nucleopsin RNA kit (Takara Bio), according to the manufacturer’s instructions. DNase I treatment was performed with the enzyme included in the RNA extraction kit. The quality of Total RNA was assessed using tapestation, and cDNA libraries were prepared using the NEBNext Ultra II Directional RNA Library Prep Kit for Illumina (New England BioLabs). We confirmed that the RIN value of extracted RNA from all samples was 10.0.

### Sequencing reaction and processing of sequencing data

**Fig. 1:**
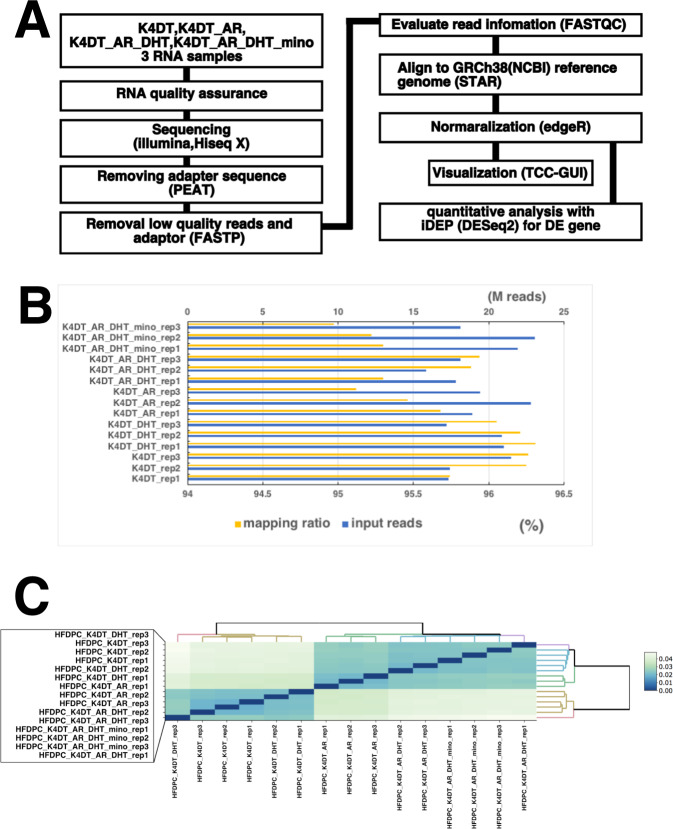
The outflow of the analysis, number of reads and mapping ratio, and correlation plots of the reads. (**A**) Outflow of the data analysis. (**B**) Number of reads for each sample and mapping ratio using STAR. (**C**) Correlation matrix of all data.

The cDNA library was created using poly-A-tailed RNA. The Hiseq X sequencer (Illumina, San Diego, CA, USA) was used to obtain pair-end 150 bp sequencing of poly-A tailed RNA. All sequencing reads were processed with PEAT to remove the adaptor sequence^6^. The low-quality reads were removed with the FASTP program^7^. The filtered reads were mapped to the NCBI reference human genome sequence (CRCh38) using the STAR program^8^. The mapped data were processed into the expression count data using featureCounts^9^ included in the Subread package. To extract differentially expressed genes (DE genes), we first performed a pairwise comparison with iDep^10^. The DE genes were detected with DESeq2^11^ with FDR cut off of 0.050 and a minimum fold change of 3. We also processed the data using TCC-GUI^12^ for Principal component analysis (PCA), heatmap analysis, and bar plot analysis (Fig. 1A).

## Data Records

### Number of reads, mapping ratio to the reference genome, and the correlation matrix for whole-genome expression counts

The quality score of reads is shown in Figures S1 to S5. The filtered reads were uploaded to the Japan DNA Database with BioProject submission ID PSUB017897 and DRA014605^13^. The number of sample reads is summarised in Fig. 1B. The number of reads per sample ranged from 16 M to 23 M, which is consistent with our previous analysis^14^. The mapping ratio to the reference genome was approximately 95 to 96.3%, which is sufficient for quantifying gene expression^5,15^. We compared the expression pattern to the correlation matrix, as shown in Fig. 1C. Interestingly, the expression pattern was completely divided into two groups, samples with no AR expression [Samples 1–3: HFDPC-K4DT (immortalized DPCs), intact condition; Samples 4–6: HFDPC-K4DT treated with 50 nM dihydroxy testosterone (DHT)] or samples with AR expression [Samples 7–9: HFDPC-K4DT with AR expression, no treatment with DHT; Samples 10–12: HFDPC-K4DT with AR expression, treated with 50 nM of DHT; Samples 13–15: HFDPC-K4DT with AR expression, treated with 50 nM DHT and 30 nM MXS]. As shown in Fig. 1C, the correlation matrix of whole expression count showed that replication 3 of HFDPC-K4DT treated with 50 nM of DHT was mixed with replication 2 and 3 of HFDPC-K4DT (immortalized DPCs), intact condition, indicating that the difference in expression profiling between Samples 1–3: HFDPC-K4DT (immortalized DPCs), intact condition, and Sample 4–6: HFDPC-K4DT (no expression of AR), treated with 50 nM DHT is very limited, suggesting that DHT treatment does not cause major expression change in the absence of AR. Furthermore, replication 1 of HFDPC-K4DT with AR expression, treated with 50 nM DHT was mixed with replication 2 and 3 of HFDPC-K4DT with AR expression, treated with 50 nM DHT and 30 nM MXS. All gene expression data were uploaded into Figshare^16^. Furthermore, the difference in expression profiling between Samples 10–12: HFDPC-K4DT with AR expression, treated with 50 nM DHT and Samples 13–15: HFDPC-K4DT with AR expression, treated with 50 nM DHT and 30 nM MXS was also very limited, indicating that MXS does not significantly influence the signal activation of AR.

### Identification of downstream genes of AR

**Fig. 2:**
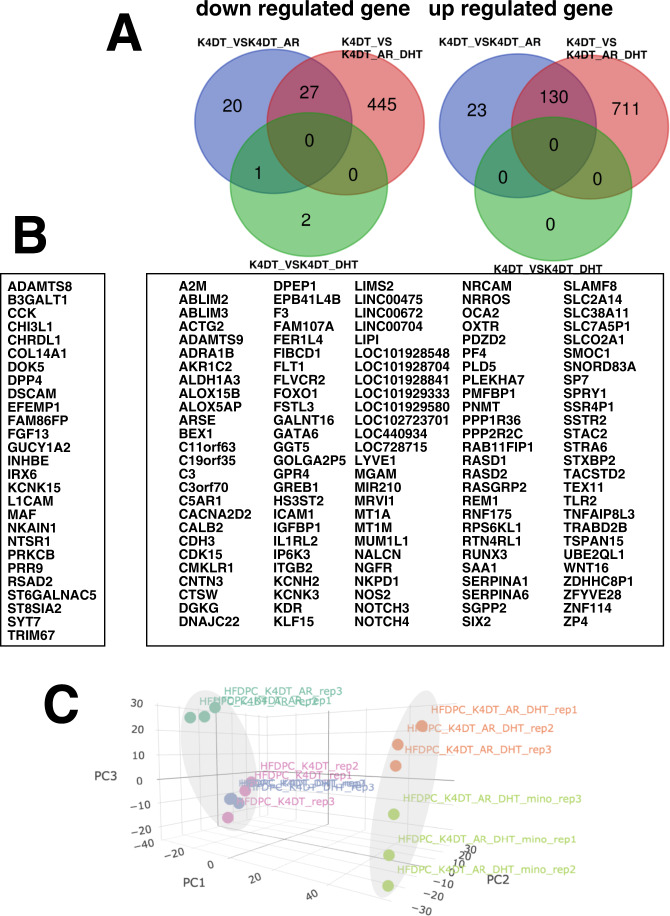
Identification of commonly downregulated and upregulated genes in AR-expressing DPCs in the presence of DPC ligand, dihydroxy testosterone (DHT). (**A**) (Left) Venn-diagram of downregulated genes in AR-expressing immortalized DPC (K4DT) and AR-expressing K4DT treated with DHT. We subtracted downregulated genes following DHT treatment in the absence of AR. (Right) Venn-diagram of upregulated genes in AR-expressing immortalized DPC (K4DT) and AR-expressing K4DT treated with DHT. We subtracted upregulated genes following DHT treatment in the absence of AR. (**B**) (Left) 27 commonly downregulated genes in AR-expressing immortalized DPC (K4DT) and AR-expressing K4DT treated with DHT detected by DESeq2 under conditions of 0.050 FDR cut off and a minimum fold change of 3 times. (Right) 130 commonly upregulated genes in AR-expressing immortalized DPC (K4DT) and AR-expressing K4DT treated with DHT detected by DESeq2 under conditions of 0.050 FDR cut off and a minimum fold change of 3 times. (**C**) Thee-dimensional Principal Component Analysis (PCA) with 25000 genes. The left side cluster contains immortalized DPCs with no AR expression and no DHT treatment, immortalized DPCs with no AR expression and treated with 50 nM DHT, and immortalized DPCs with AR expression and no DHT treatment. The right-side cluster contains immortalized DPCs with AR expression and DHT treatment and immortalized DPCs with AR expression and treated with DHT and minoxidil sulphate (MXS) treatment.

We performed a pairwise comparison of Samples 1–3: HFDPC-K4DT and Samples 7–9: HFDPC-K4DT with AR expression with no DHT treatment with Dseq2 using iDep. As shown in the left panel of Fig. 2, blue circle, 48 DE genes were identified (20 + 1 + 27 + 0 genes, blue circle of Fig. 2A, left panel) as downregulated genes in HFDPC-K4DT with AR expression (control is HFDPC-K4DT). Furthermore, 153 DE genes were identified as upregulated genes (23 + 130 + 0 + 0 genes, blue circle of Fig. 2A, right panel). We used Dseq2 to perform a pairwise comparison between Sample 1–3: HFDPC-K4DT and Samples 10–12: HFDPC-K4DT with AR expression, treated with 50 nM DHT. As shown in the left panel of Fig. 2, orange circle, 472 genes were identified as downregulated genes in HFDPC-K4DT with AR expression, treated with 50 nM DHT, (445 + 27 + 0 + 0 genes, orange circle of Fig. 2A, left panel). As shown in the right panel of Fig. 2, orange circle, 841 DE genes were identified as upregulated genes (711 + 130 + 0 + 0 genes, orange circle of Fig. 2A, right panel). We predicted that true downstream genes of AR signalling would be commonly upregulated or downregulated, though the degree of gene expression might differ with or without DHT ligand treatment. From this viewpoint, we counted the number of genes that were commonly downregulated or upregulated genes between the blue and orange circles in Fig. 2A. As shown, 27 genes were identified as downregulated (overlap of the blue and orange circle). The overlap region also revealed 130 genes that were upregulated. Furthermore, we compared Samples 1–3: HFDPC-K4DT and Samples 4–6: HFDPC-K4DT, treated with 50 nM DHT-No expression of AR. Because cells of Samples 1–3 and Samples 4–6 do not express AR, if genes in these samples are downregulated or upregulated following DHT ligand treatment, it indicates that these DE genes are affected through an independent pathway or receptor other than AR. As shown in Fig. 2A, three genes were identified as downregulated (Fig. 2A, left side, green circle), whereas no gene was upregulated (Fig. 2A, right side, green circle). Although we tried to subtract those green circle genes from the overlap region of green and orange genes (commonly down or up-regulated genes), there was no overlap between them. Based on these findings, we listed 27 downregulated genes and 130 upregulated genes as potential candidates for downstream genes of AR signalling pathways (Fig. 2B).

### PCA of gene expression

We performed PCA on gene expression profiling. Due to software limitation, the top 25,000 genes were the maximum number of genes involved in the analysis. As demonstrated by the results of 3D-PCA in Fig. 2C and Movie S1^17^, experimental groups were primarily classified into two clusters: cell cluster with DHT treatment and cell cluster without DHT treatment (Fig. 2C, left side and right side, respectively). These results indicate that ligand stimulation by DHT induces a significant change in expression profiling. Within the cell cluster without DHT (Fig. 2C, left side), Samples 7–9: HFDPC-K4DT with AR expression, no treatment with DHT formed a sub-cluster that was relatively distant from Samples 1–3: HFDPC-K4DT (immortalized DPCs), intact condition, and Samples 4–6: HFDPC-K4DT treated with 50 nM of DHT, no AR expression.

### Heatmap analysis of downstream genes of AR

**Fig. 3:**
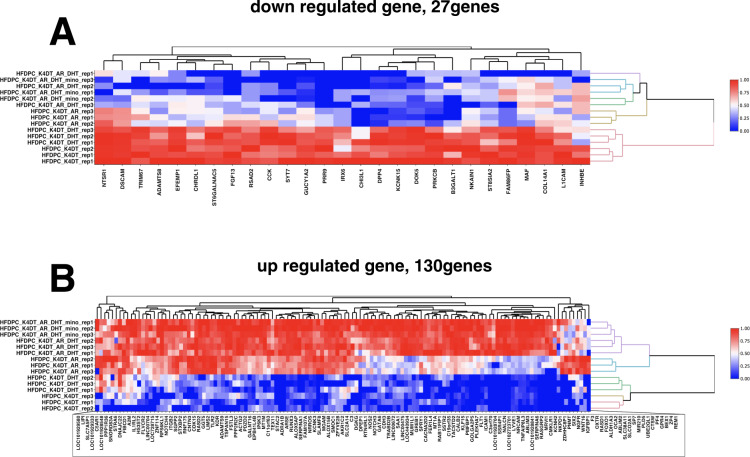
Heat map of the 27 downregulated and 130 upregulated genes. Red colour represents high expression and blue colour represents low expression of corresponding genes. (**A**) the Heat map of the 27 downregulated genes. Note that most of the genes listed in this panel showed high expression levels in immortalized DPCs without AR expression and no DHT treatment. Furthermore, even after DHT treatment, expression patterns look almost identical in the absence of AR (HFDPC_K4DT_DHT_rep1–3).

As shown in Fig. 3A, the pairwise analysis identified the expression levels of 27 genes. Samples 1–3: HFDPC-K4DT (immortalized DPCs), intact condition, and Sample 4–6: HFDPC-K4DT treated with 50 nM DHT, no expression of AR had higher expression of 27 genes. However, Samples 10–12: HFDPC-K4DT with AR expression treated with 50 nM DHT and Samples 13–15: HFDPC-K4DT with AR expression treated with 50 nM DHT and 30 nM MXS exhibited lower expression level of these genes.

Furthermore, we analysed the 130 upregulated genes. As shown in Fig. 3B, gene expression levels were lower in Samples 1–3: HFDPC-K4DT (immortalized DPCs), intact condition, and Samples 4–6: HFDPC-K4DT treated with 50 nM DHT, no AR expression. However, Samples 10–12: HFDPC-K4DT with AR expression treated with 50 nM DHT and Samples 13–15: HFDPC-K4DT with AR expression treated with 50 nM DHT and 30 nM MXS exhibited higher expression level of listed genes. Furthermore, Samples 7–9: HFDPC-K4DT with AR expression, no DHT treatment showed an expression pattern around the midpoint between the sample without DHT treatment and the sample with DHT treatment.

### Expression count of potential candidates of AR downstream genes

The bar plots of the downregulated 27 candidates are listed in Figures S6–S8. For reliable and reproducible further analysis, such as RT-PCR, at least 1000 expression counts are required. From this viewpoint, DPP4, KCNK15, and NTSR1 were listed as downregulated candidates. The bar plots of upregulated 130 genes are listed in Figures S9 to S24. We filtered 1000 expression counts of corresponding data. The candidates, ABLIM3, AKR1C2, EPB41L4B, FSTL3, ICAM1, IGFBP1, LYVE1, MT1M, NRCAM, OXTR, SLAMF8, TNFAIP8L3, and WNT16 are listed. Our data sets would be an ideal material to identify the expression change of known and unknown downstream genes of AR signalling. Furthermore, the data are of great use to scientists working on the effect of AR signalling on prostate cancers and AGA.

### Identification of differentially expressed non-coding RNAs

**Fig. 4:**
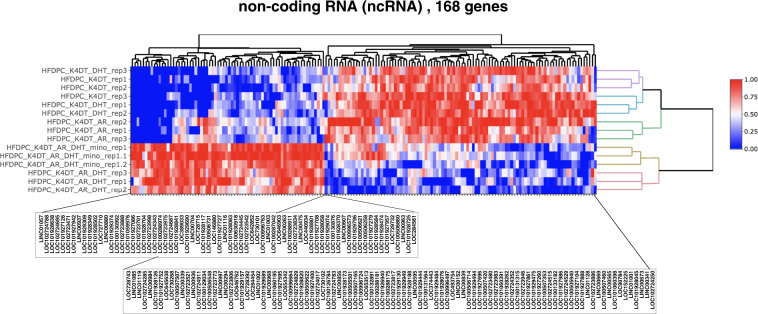
Heat map of 168 differentially expressed non-coding RNAs. Red colour represents high expression and blue colour represents low expression of corresponding genes.

Non-coding RNAs such as lncRNA was reported to control the transcriptional activity of promoter region and gene expression. To elucidate the differentially expressed lncRNA in our study, we identified differentially expressed lnc RNAs. As shown in Fig. 4, we identified that 168 RNAs. The expression pattern of lncRNAs were completely separated among activated samples of AR pathway and inactivated sample.

## Technical Validation

### RNA Quality

The RNA quality data was submitted to Figshare^18^. The RIN value of all the RNA was 10.0, indicating that the sample we obtained was in near-perfect condition.

### Mapping ratio and number of reads, reproducibility of the biological replications, and experimental controls

To evaluate the reproducibility of the data, triple biological replicates were established. The setup of experimental groups is described in the method section. We established AR negative cells and positive cells. Similarly, we established DHT ligand-treated cells and untreated cells. In addition to these groups, immortalized DPCs without AR expression and treated by DHT ligands were established. The upregulated or downregulated genes in these groups suggest that detected gene expression change is mediated by a pathway other than the AR pathway.

## Supplementary information

Fig_S1_S24

## Data Availability

We have listed the names and versions of the software used for data analysis. FastQC, version 0.11.3, was used for the quality check of the raw FASTQ sequencing file. https://www.bioinformatics.babraham.ac.uk/projects/fastqc/. FASTP, version 0.23.1, was used to remove low-quality reads. https://github.com/OpenGene/fastp#per-read-cutting-by-quality-score. PEAT, version 1.2, was used to remove the adaptor sequence. https://github.com/jhhung/PEAT. STAR, version 2.6.1 was used for mapping. https://github.com/alexdobin/STAR. featureCount, SUBREAD, release 1.6.5 was used for expression counting. http://subread.sourceforge.net R package, version 4.0.3, was used for downstream gene analysis. https://www.r-project.org iDEP was used for the identification of DE genes. http://bioinformatics.sdstate.edu/idep/ TCC-GUI tool was used for downstream gene analysis. https://github.com/swsoyee/TCC-GUI.
