# Supplementary material for: Comprehensive transcriptome data to identify downstream genes of testosterone signalling in dermal papilla cells
Source: Sci Data. 2022 Nov 29;9:731. doi: 10.1038/s41597-022-01846-w (PMC9709033; doi:10.1038/s41597-022-01846-w)
Supplement: Supplementary file 1 — Fig_S1_S24 [file 41597_2022_1846_MOESM1_ESM.pdf]

HFDPC-K4DT-1-R1

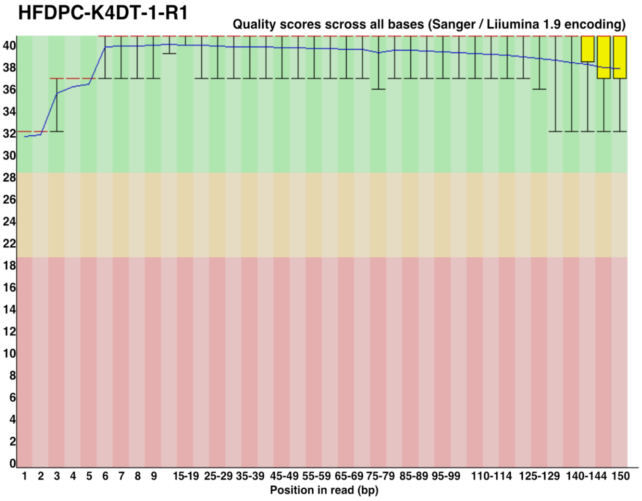

HFDPC-K4DT-1-R2

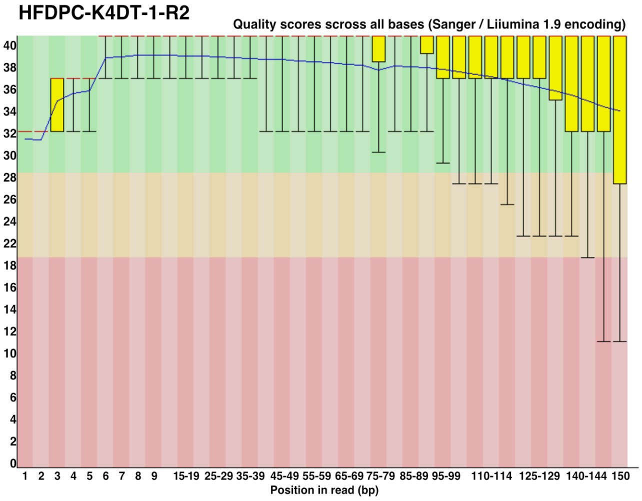

HFDPC-K4DT-2-R1

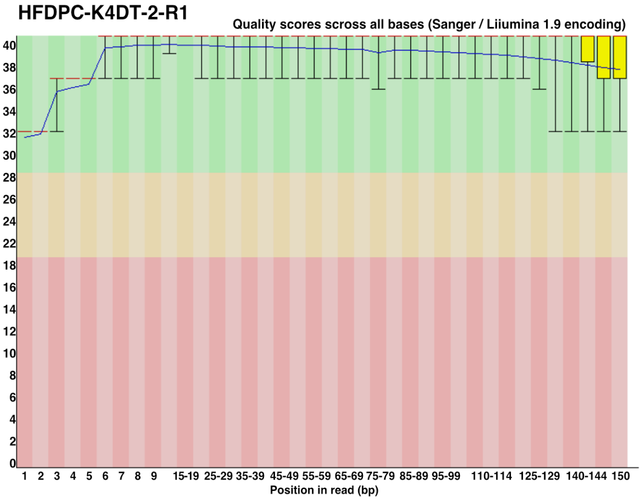

HFDPC-K4DT-2-R2

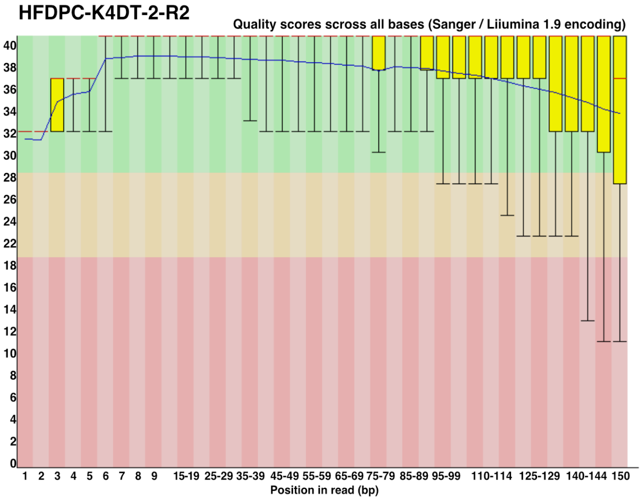

HFDPC-K4DT-3-R1

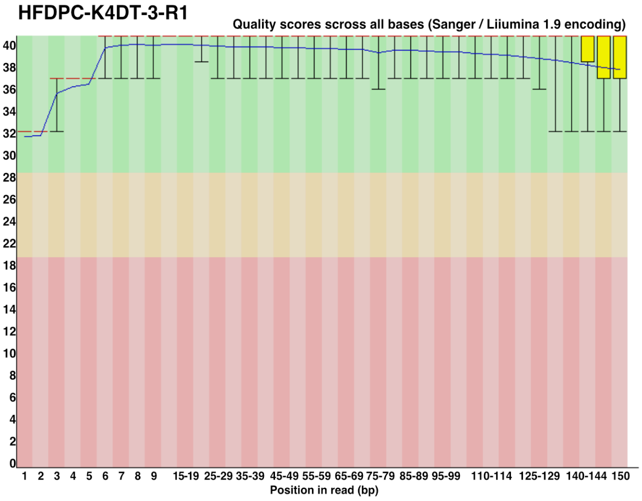

HFDPC-K4DT-3-R2

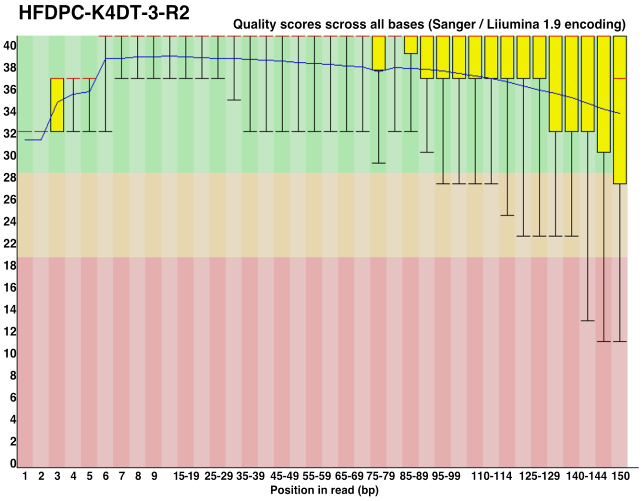

Figure S1. Quality mapping results of sequencing data. R1 and R2 reads means paired-end.

HFDPC-K4DT-DHT-1-R1

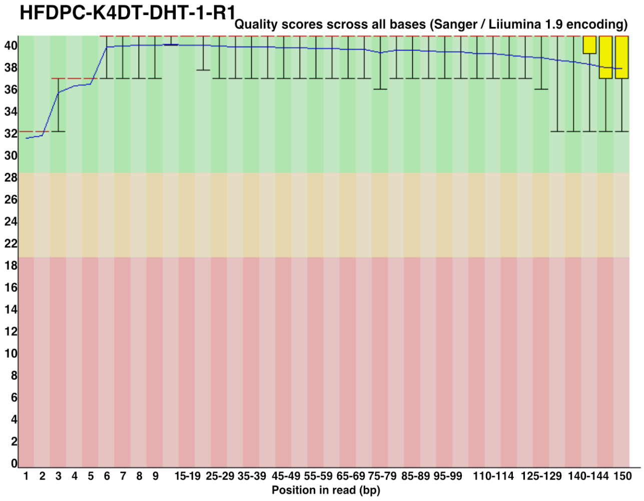

HFDPC-K4DT-DHT-1-R2

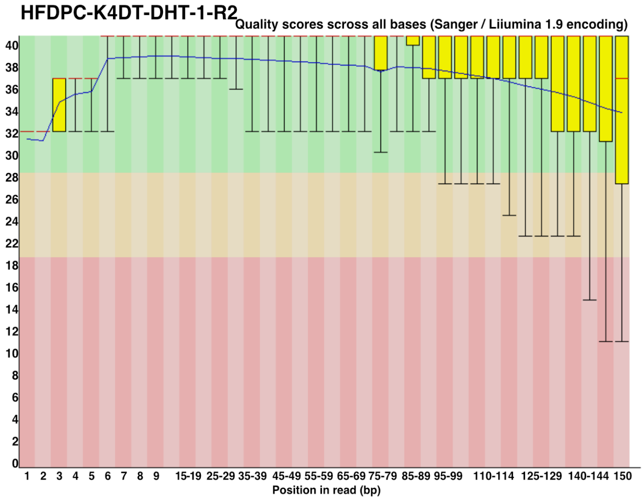

HFDPC-K4DT-DHT-2-R1

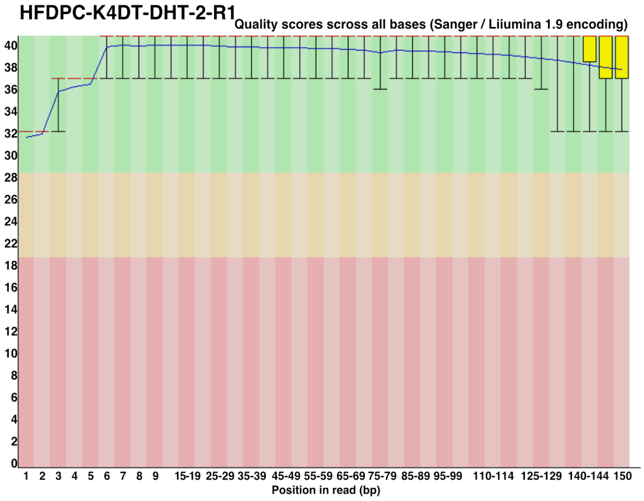

HFDPC-K4DT-DHT-2-R2

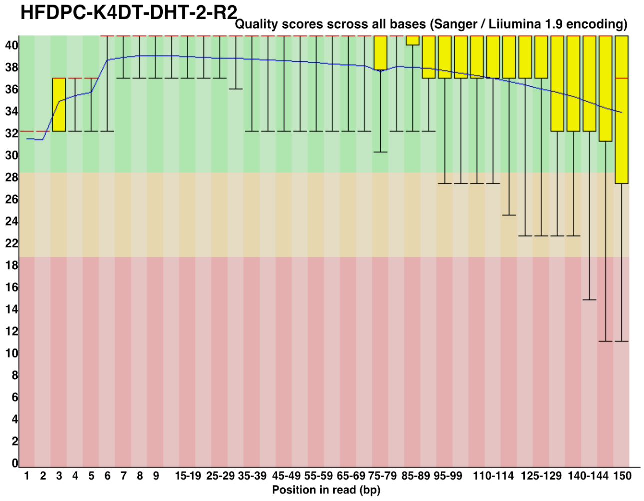

HFDPC-K4DT-DHT-3-R1

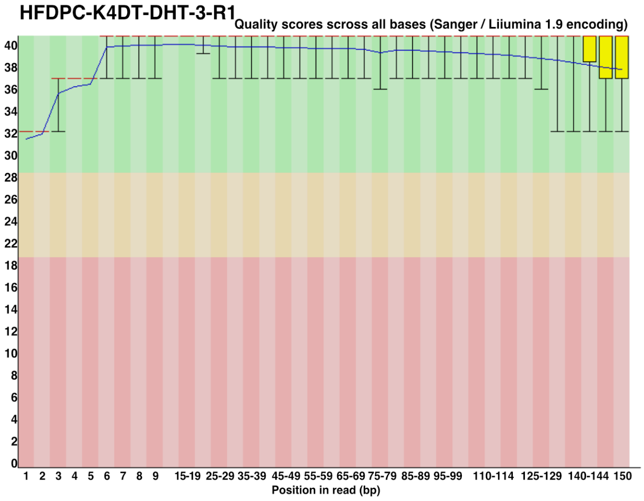

HFDPC-K4DT-DHT-3-R2

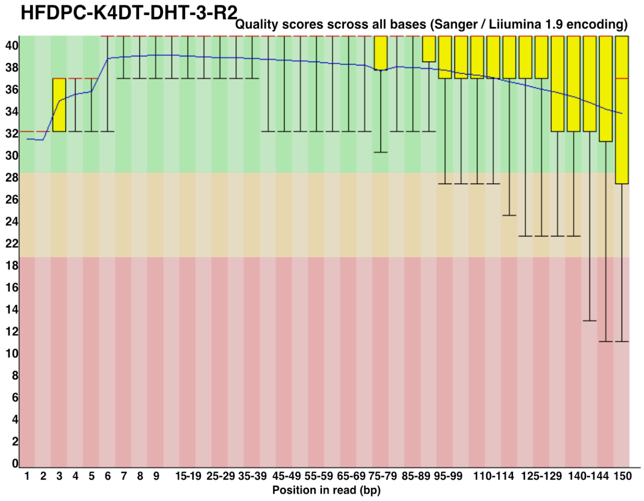

Figure S2. Quality mapping results of sequencing data. R1 and R2 reads means paired-end.

HFDPC-K4DT-AR-1-R1

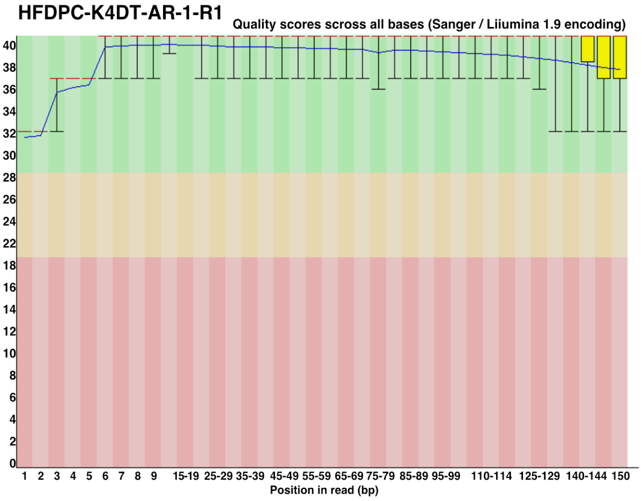

HFDPC-K4DT-AR-1-R2

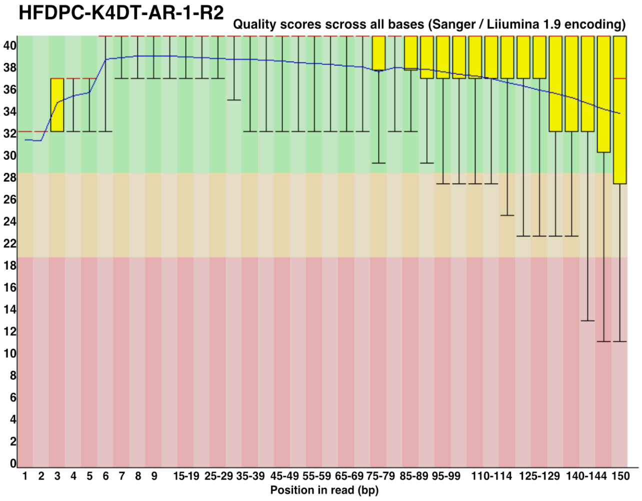

HFDPC-K4DT-AR-2-R1

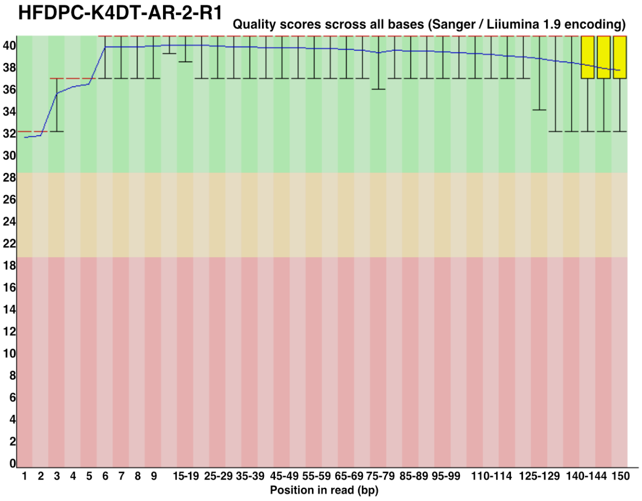

HFDPC-K4DT-AR-2-R2

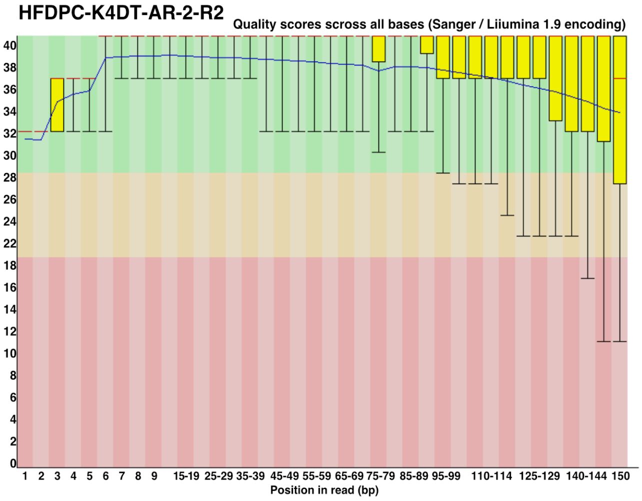

HFDPC-K4DT-AR-3-R1

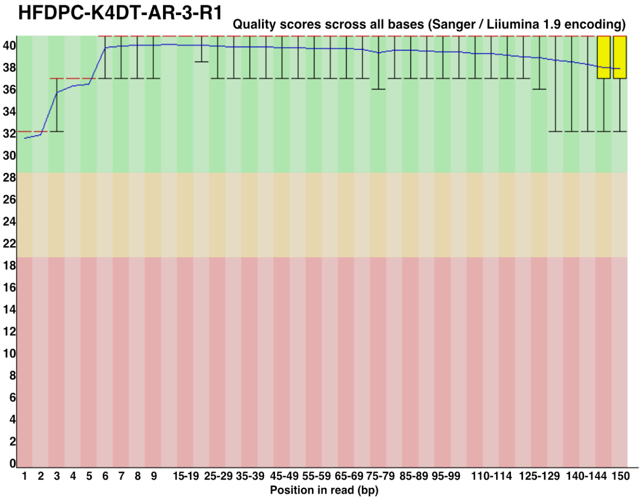

HFDPC-K4DT-AR-3-R2

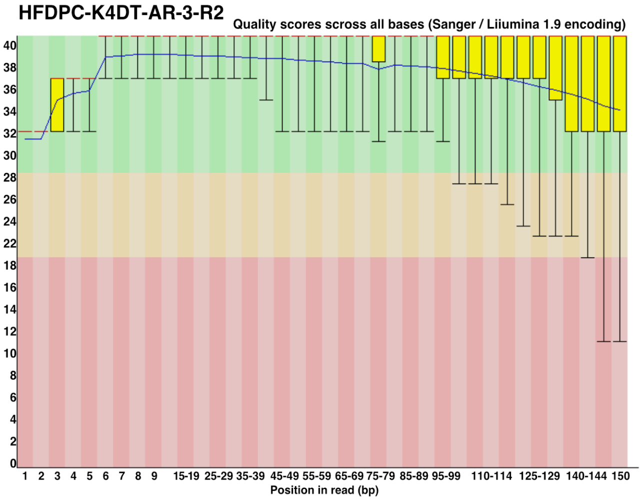

Figure S3. Quality mapping results of sequencing data. R1 and R2 reads means paired-end.

HFDPC-K4DT-AR-DHT-1-R1

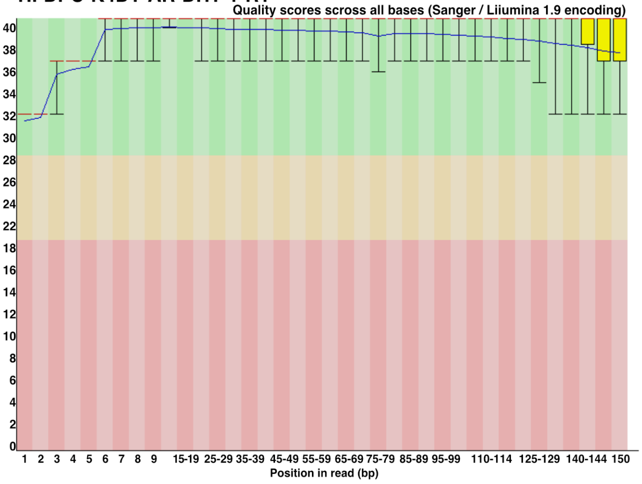

HFDPC-K4DT-AR-DHT-1-R2

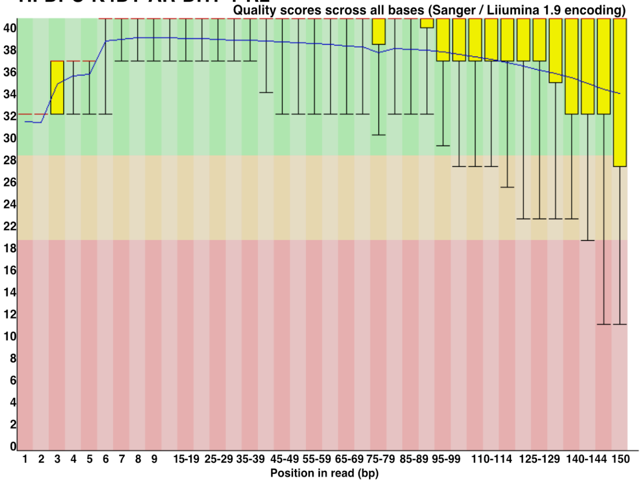

HFDPC-K4DT-AR-DHT-2-R1

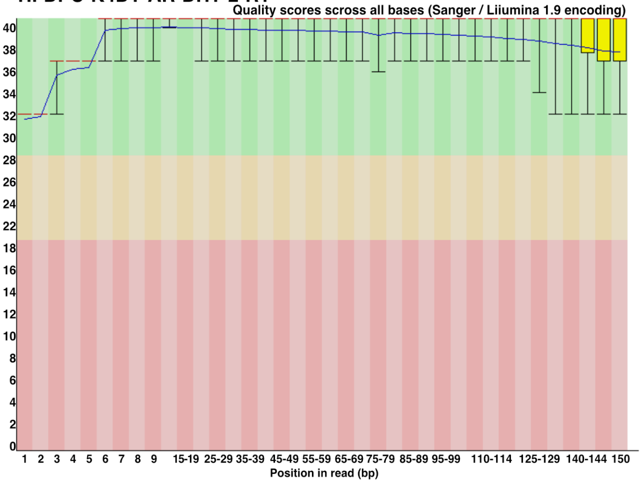

HFDPC-K4DT-AR-DHT-2-R2

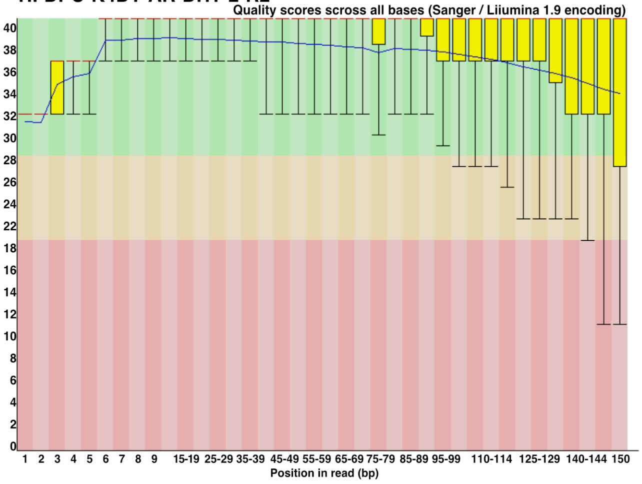

HFDPC-K4DT-AR-DHT-3-R1

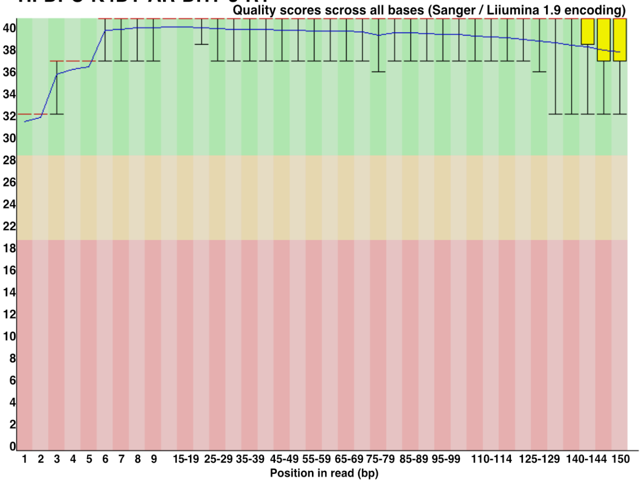

HFDPC-K4DT-AR-DHT-3-R2

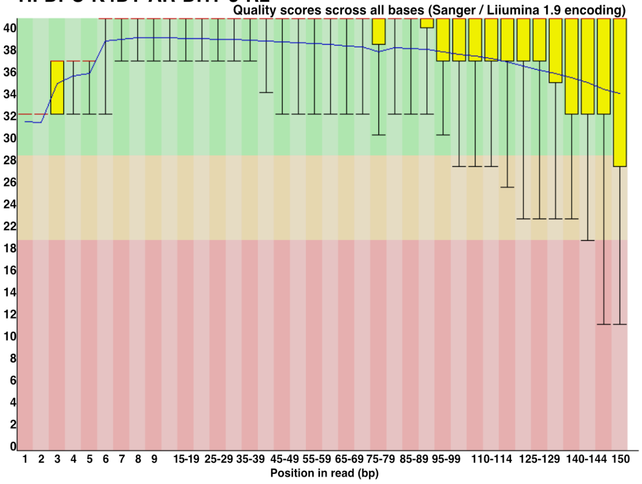

Figure S4. Quality mapping results of sequencing data. R1 and R2 reads means paired-end.

HFDPC-K4DT-AR-DHT-mino-1-R1

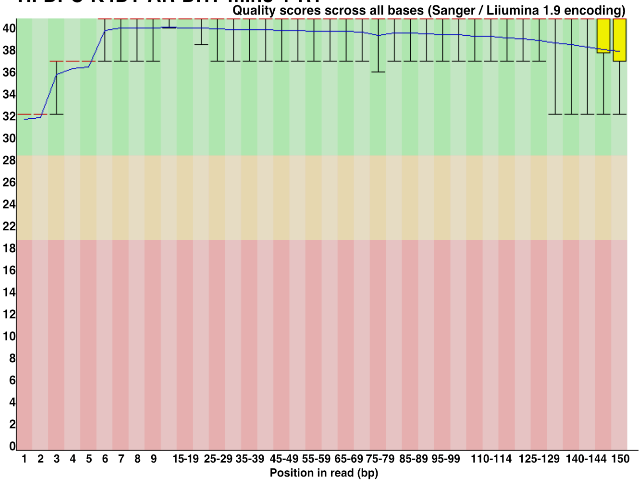

HFDPC-K4DT-AR-DHT-mino-1-R2

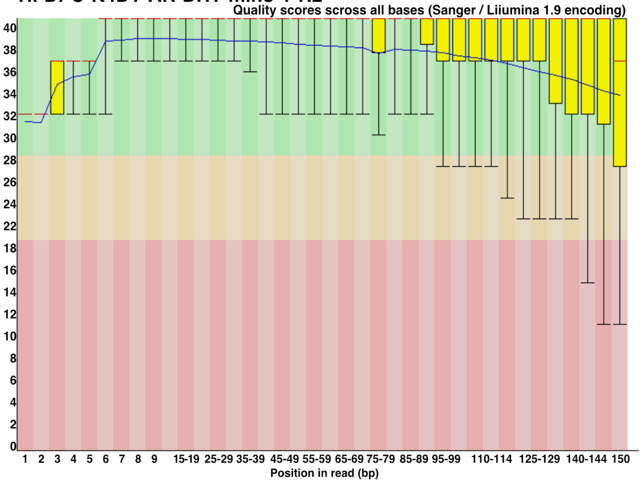

HFDPC-K4DT-AR-DHT-mino-2-R1

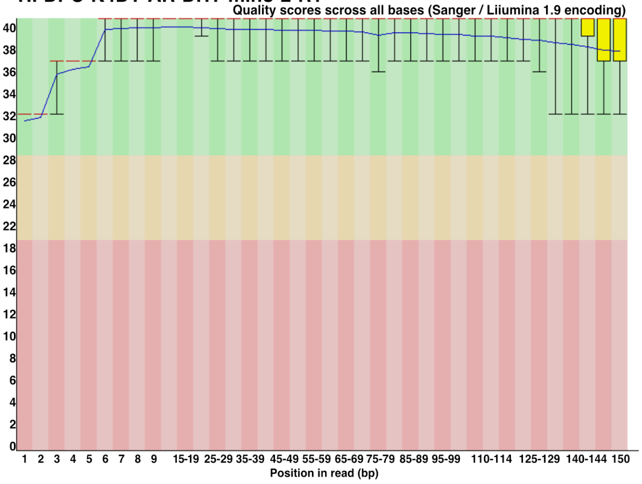

HFDPC-K4DT-AR-DHT-mino-2-R2

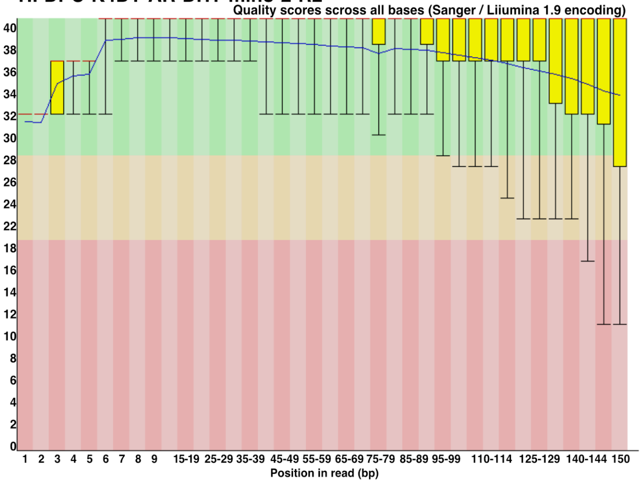

HFDPC-K4DT-AR-DHT-mino-3-R1

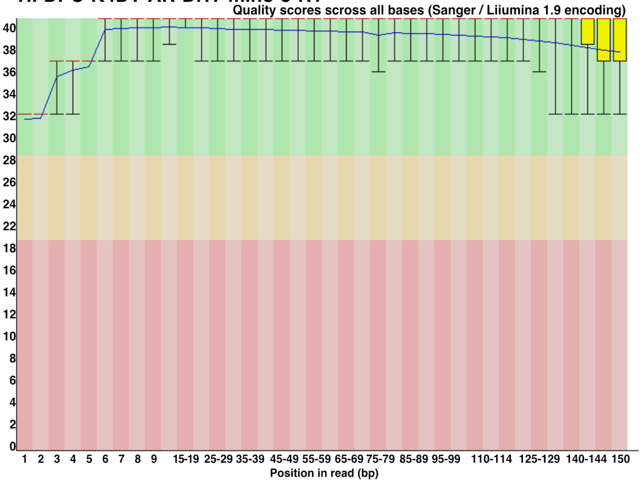

HFDPC-K4DT-AR-DHT-mino-3-R2

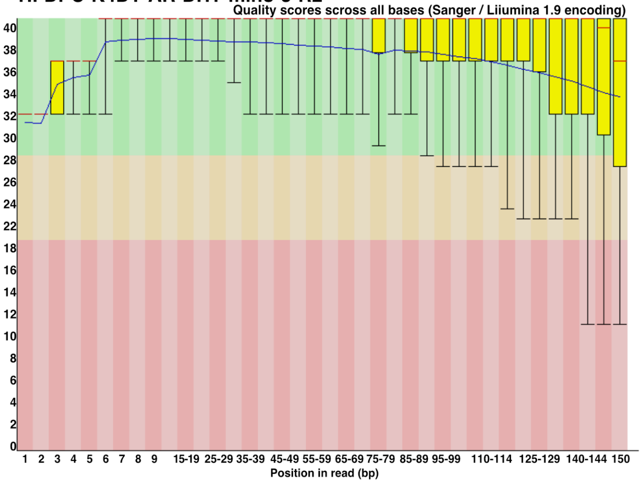

Figure S5. Quality mapping results of sequencing data. R1 and R2 reads means paired-end.

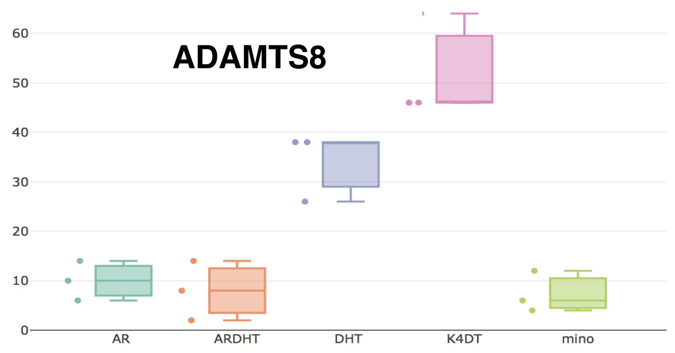
ADAMTS8
60
50
40
30
20
10
0
AR
ARDHT
DHT
K4DT
mino

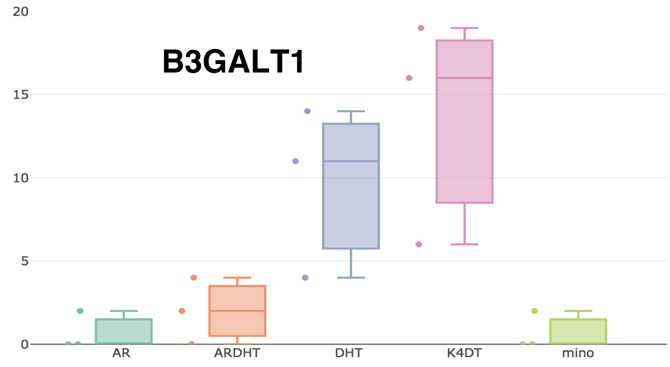
B3GALT1
20
15
10
5
0
AR
ARDHT
DHT
K4DT
mino

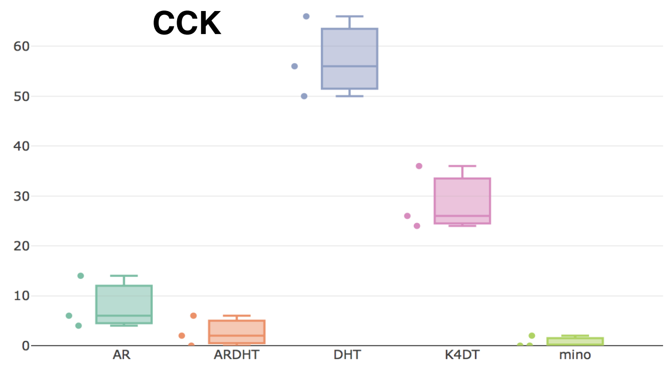
CCK
60
50
40
30
20
10
0
AR
ARDHT
DHT
K4DT
mino

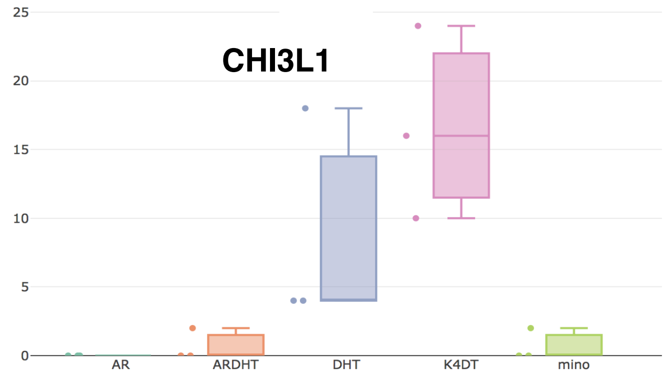
CHI3L1
25
20
15
10
5
0
AR
ARDHT
DHT
K4DT
mino

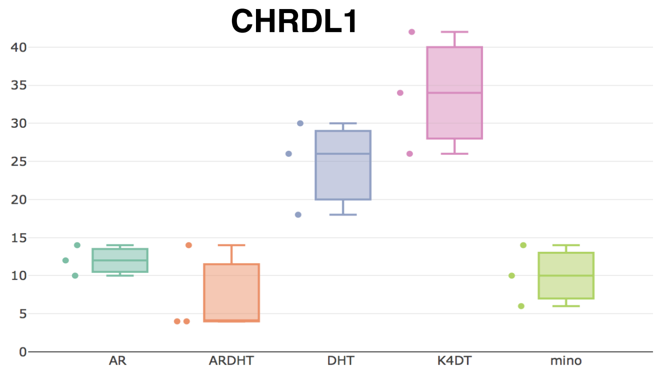
CHRDL1
40
35
30
25
20
15
10
5
0
AR
ARDHT
DHT
K4DT
mino

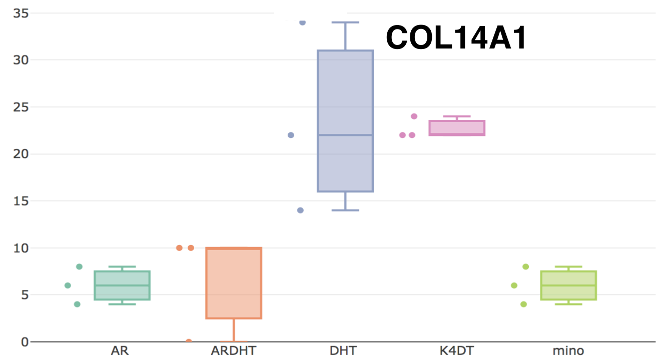
COL14A1
35
30
25
20
15
10
5
0
AR
ARDHT
DHT
K4DT
mino

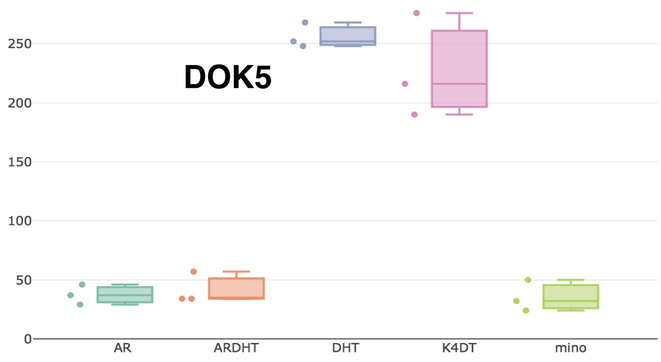
DOK5
250
200
150
100
50
0
AR
ARDHT
DHT
K4DT
mino

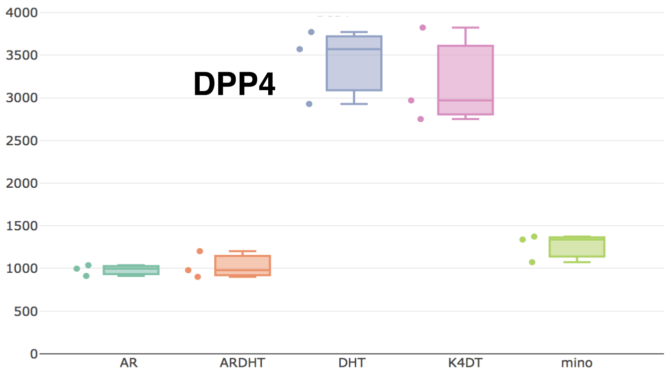
DPP4
4000
3500
3000
2500
2000
1500
1000
500
0
AR
ARDHT
DHT
K4DT
mino

Figure S6. Bar plot mapping of 27 downregulated genes. Results of ADAMTS8, B3GALT1, CCK, CHI3L1, CHRDL1, COL14A1, DOK5, DPP4 were shown. AR is HFDPC with AR expression and no ligand treatment. ARDHT is HFDPC with AR expression and ligand treated. DHT is HFDPC without AR expression and ligand treated. K4DT is HFDPC without AR expression and no ligand treatment. Mino is HFDPC with AR expression and ligand and Minoxidil sulfate (MXS) treated.

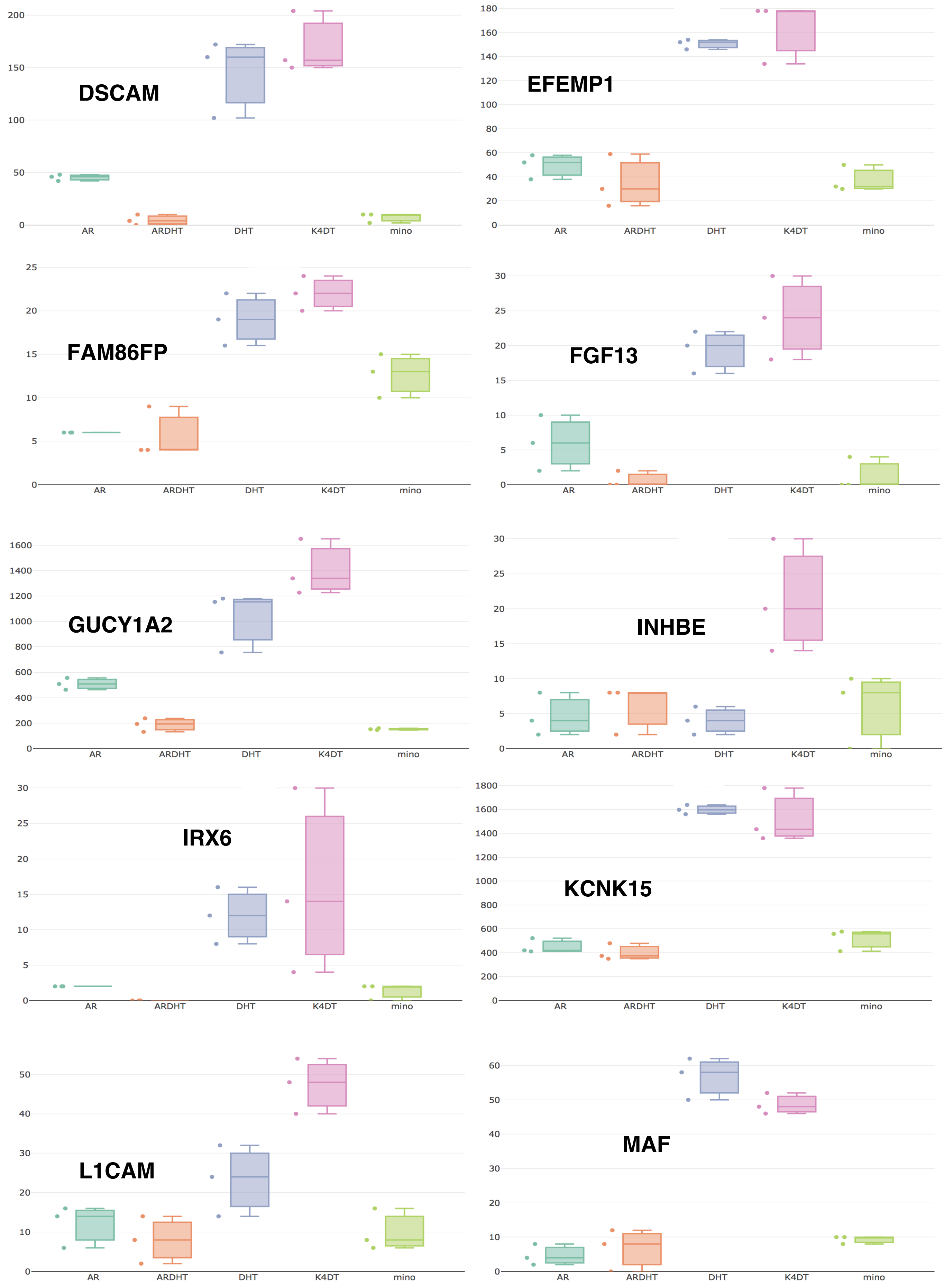
DSCAM
EFEMP1
FAM86FP
FGF13
GUCY1A2
INHBE
IRX6
KCNK15
L1CAM
MAF
AR
ARDHT
DHT
K4DT
mino

Figure S7. Bar plot mapping of 27 downregulated genes. Results of DSCAM, EFEMP1, FAM86FP, FGF13, GUCY1A2, INHBE, IRX6, KCNK15, L1CAM, MAF were shown. AR is HFDPC with AR expression and no ligand treatment. ARDHT is HFDPC with AR expression and ligand treated. DHT is HFDPC without AR expression and ligand treated. K4DT is HFDPC without AR expression and no ligand treatment. Mino is HFDPC with AR expression and ligand and Minoxidil sulfate (MXS) treated.

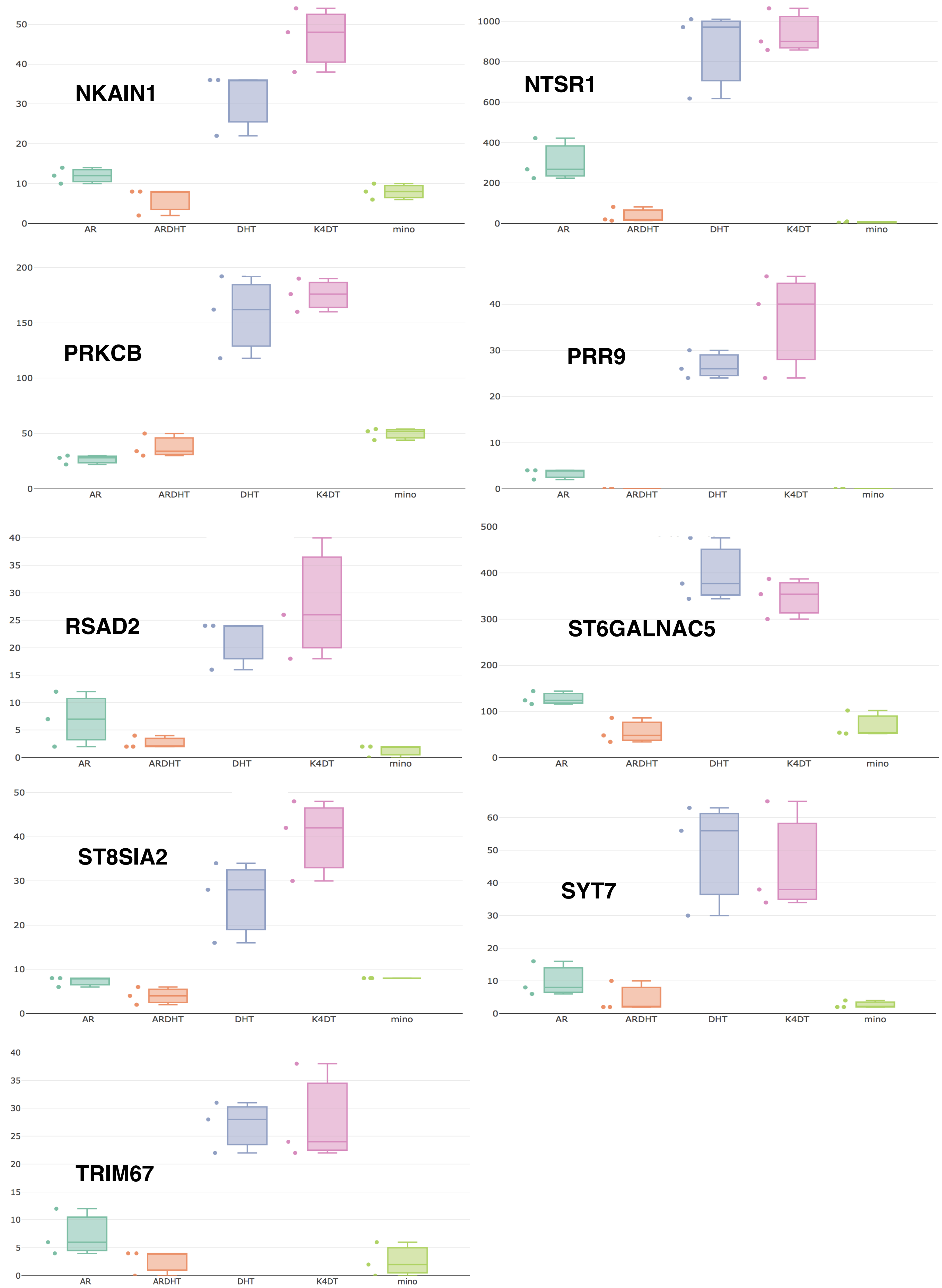
NKAIN1
0
10
20
30
40
50
AR
ARDHT
DHT
K4DT
mino
NTSR1
0
200
400
600
800
1000
AR
ARDHT
DHT
K4DT
mino
PRKCB
0
50
100
150
200
AR
ARDHT
DHT
K4DT
mino
PRR9
0
10
20
30
40
AR
ARDHT
DHT
K4DT
mino
RSAD2
0
5
10
15
20
25
30
35
40
AR
ARDHT
DHT
K4DT
mino
ST6GALNAC5
0
100
200
300
400
500
AR
ARDHT
DHT
K4DT
mino
ST8SIA2
0
10
20
30
40
50
AR
ARDHT
DHT
K4DT
mino
SYT7
0
10
20
30
40
50
60
AR
ARDHT
DHT
K4DT
mino
TRIM67
0
5
10
15
20
25
30
35
40
AR
ARDHT
DHT
K4DT
mino

Figure S8. Bar plot mapping of 27 downregulated genes. Results of NKAIN1, NTSR1, PRKCB, PRR9, RSAD2, ST6GALNAC5, ST8SIA2, SYT7, TRIM67 were shown. AR is HFDPC with AR expression and no ligand treatment. ARDHT is HFDPC with AR expression and ligand treated. DHT is HFDPC without AR expression and ligand treated. K4DT is HFDPC without AR expression and no ligand treatment. Mino is HFDPC with AR expression and ligand and Minoxidil sulfate (MXS) treated.

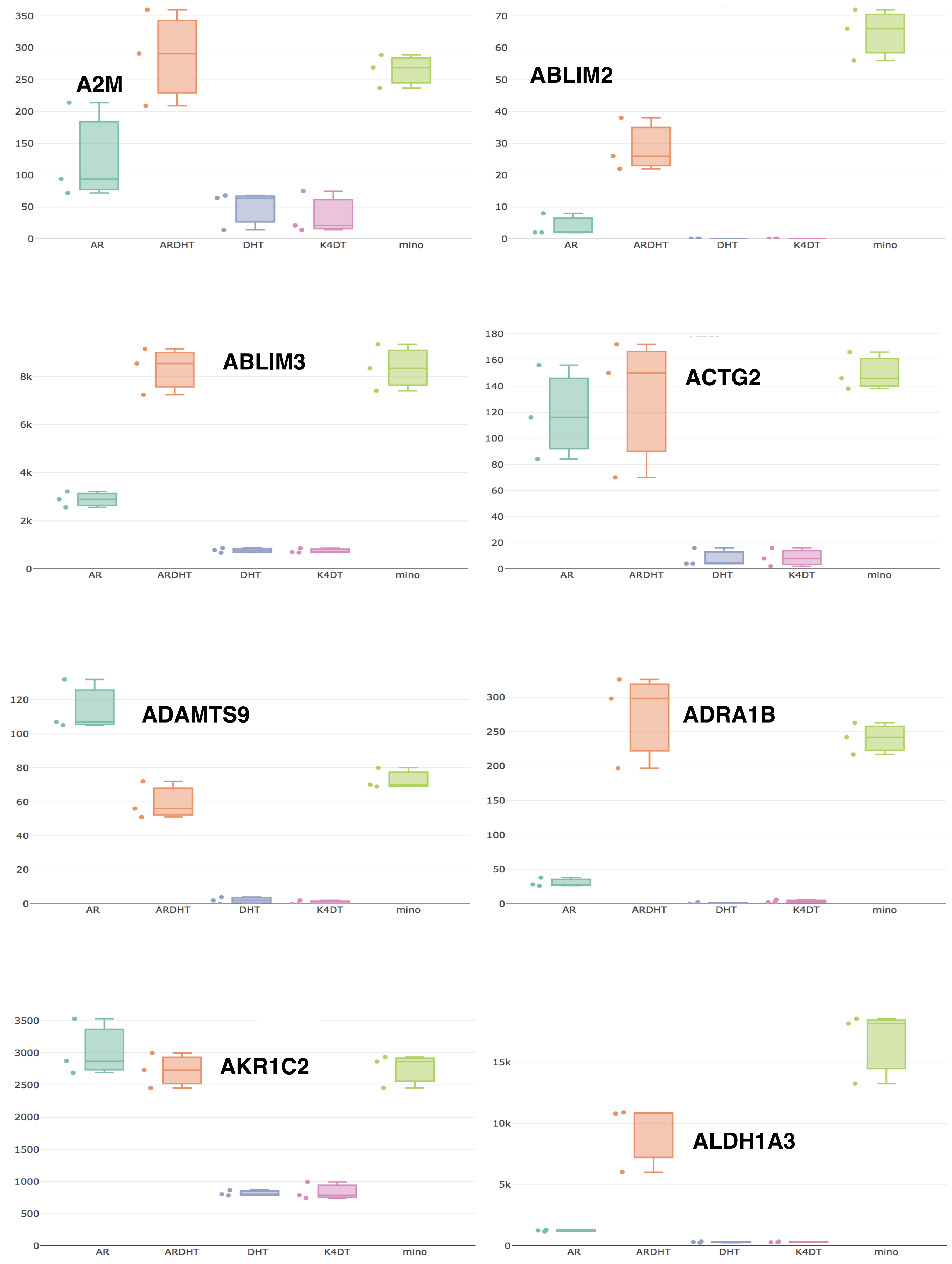
A2M
ABLIM2
ABLIM3
ACTG2
ADAMTS9
ADRA1B
AKR1C2
ALDH1A3
AR
ARDHT
DHT
K4DT
mino

Figure S9. Bar plot mapping of 130 upregulated genes. Results of A2M, ABLIM2, ABLIM3, ACTG2, ADAMTS9, ADRA1B, AKR1C2, ALDH1A3 were shown. AR is HFDPC with AR expression and no ligand treatment. ARDHT is HFDPC with AR expression and ligand treated. DHT is HFDPC without AR expression and ligand treated. K4DT is HFDPC without AR expression and no ligand treatment. Mino is HFDPC with AR expression and ligand and Minoxidil sulfate (MXS) treated.

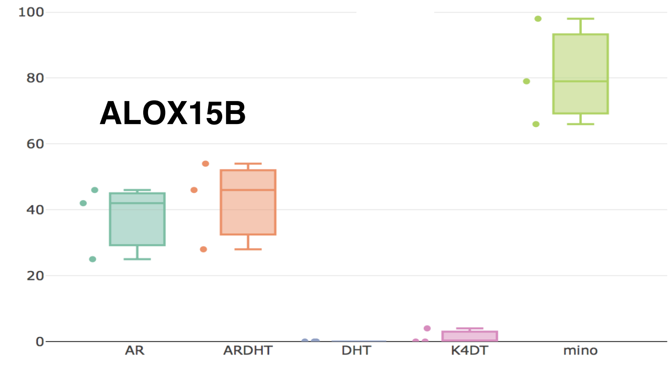
ALOX15B
0
20
40
60
80
100
AR
ARDHT
DHT
K4DT
mino

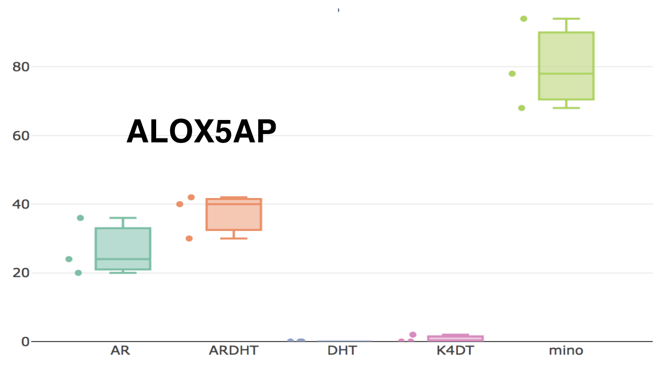
ALOX5AP
0
20
40
60
80
AR
ARDHT
DHT
K4DT
mino

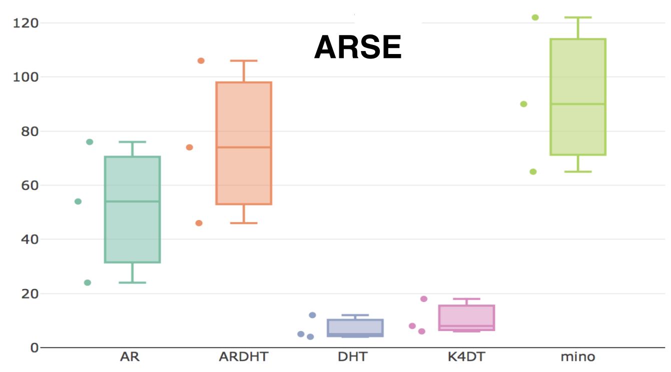
ARSE
0
20
40
60
80
100
120
AR
ARDHT
DHT
K4DT
mino

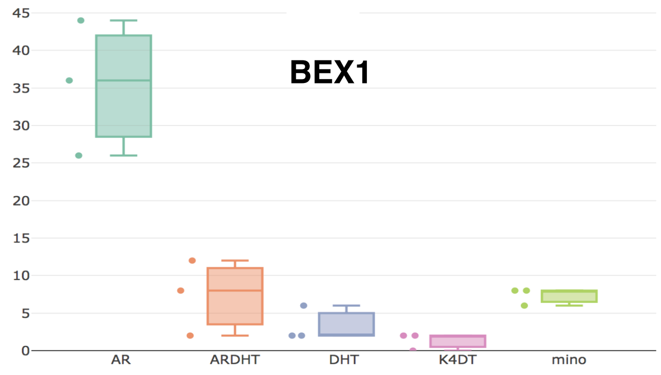
BEX1
0
5
10
15
20
25
30
35
40
45
AR
ARDHT
DHT
K4DT
mino

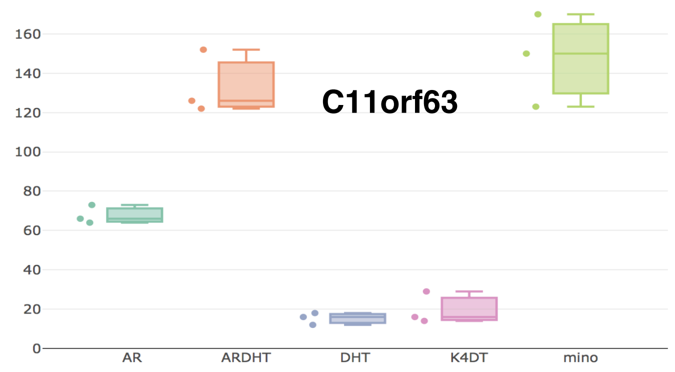
C11orf63
0
20
40
60
80
100
120
140
160
AR
ARDHT
DHT
K4DT
mino

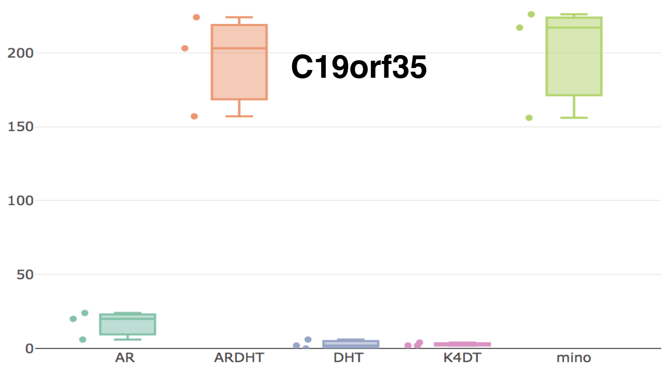
C19orf35
0
50
100
150
200
AR
ARDHT
DHT
K4DT
mino

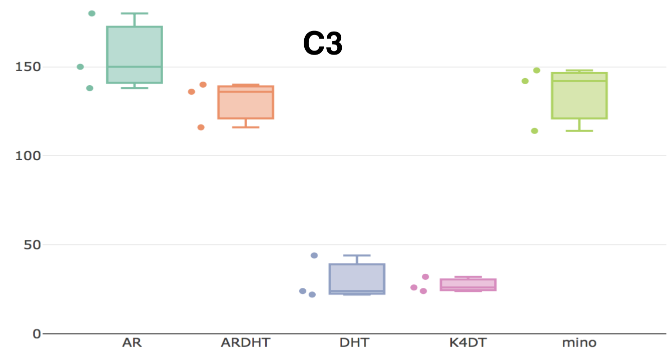
C3
0
50
100
150
AR
ARDHT
DHT
K4DT
mino

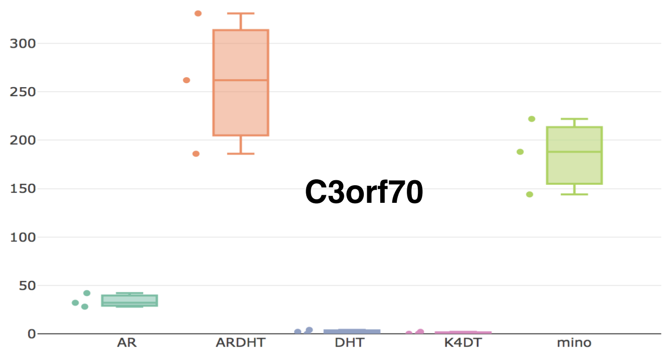
C3orf70
0
50
100
150
200
250
300
AR
ARDHT
DHT
K4DT
mino

Figure S10. Bar plot mapping of 130 upregulated genes. Results of ALOX15B, ALOX5AP, ARSE, BEX1, C11orf63, C19orf35, C3, C3orf70 were shown. AR is HFDPC with AR expression and no ligand treatment. ARDHT is HFDPC with AR expression and ligand treated. DHT is HFDPC without AR expression and ligand treated. K4DT is HFDPC without AR expression and no ligand treatment. Mino is HFDPC with AR expression and ligand and Minoxidil sulfate (MXS) treated.

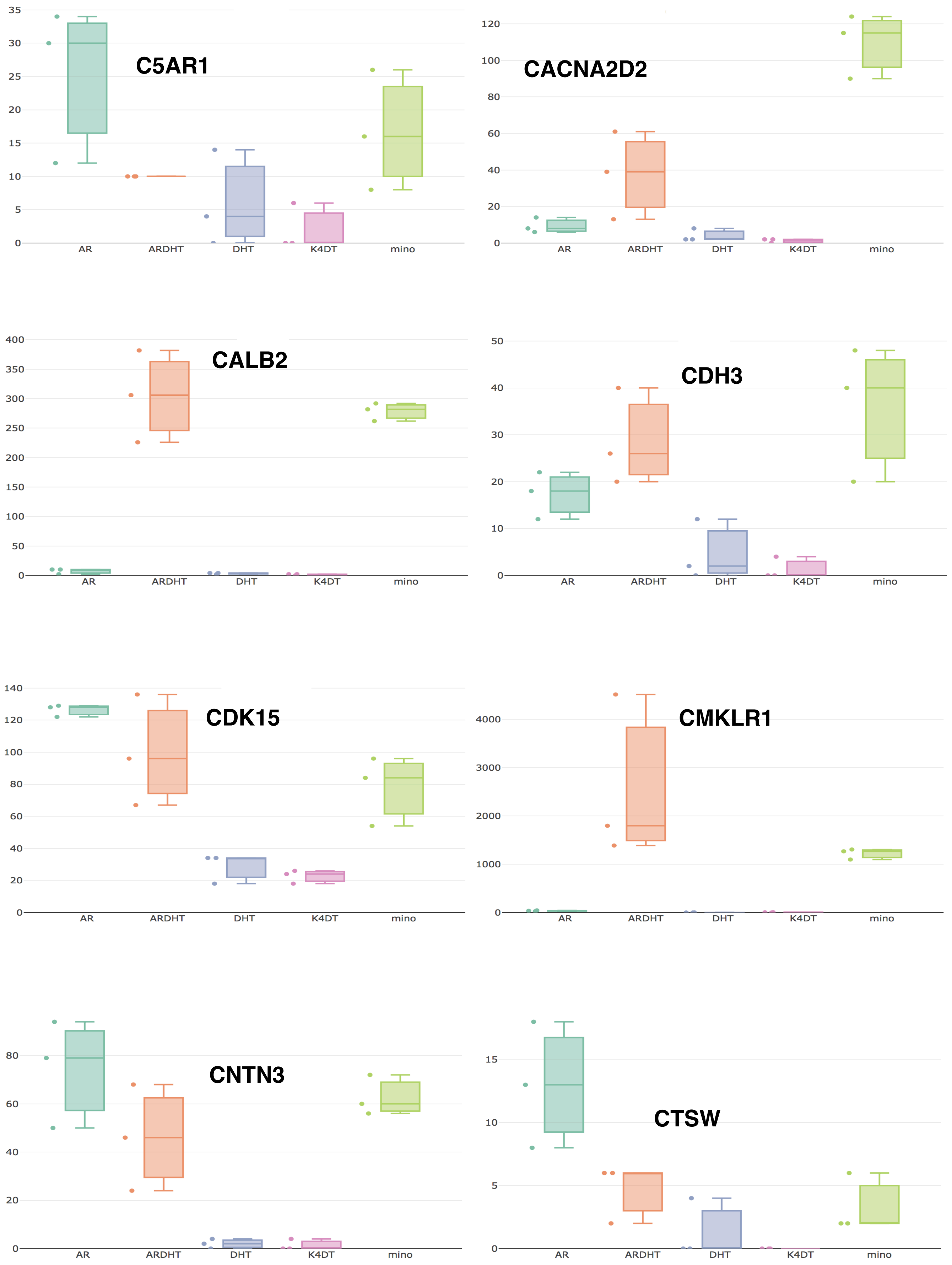
C5AR1
CACNA2D2
CALB2
CDH3
CDK15
CMKLR1
CNTN3
CTSW
AR
ARDHT
DHT
K4DT
mino

Figure S11. Bar plot mapping of 130 upregulated genes. Results of C5AR1, CACNA2D2, CALB2, CDH3, CDK15, CMKLR1, CNTN3, CTSW were shown. AR is HFDPC with AR expression and no ligand treatment. ARDHT is HFDPC with AR expression and ligand treated. DHT is HFDPC without AR expression and ligand treated. K4DT is HFDPC without AR expression and no ligand treatment. Mino is HFDPC with AR expression and ligand and Minoxidil sulfate (MXS) treated.

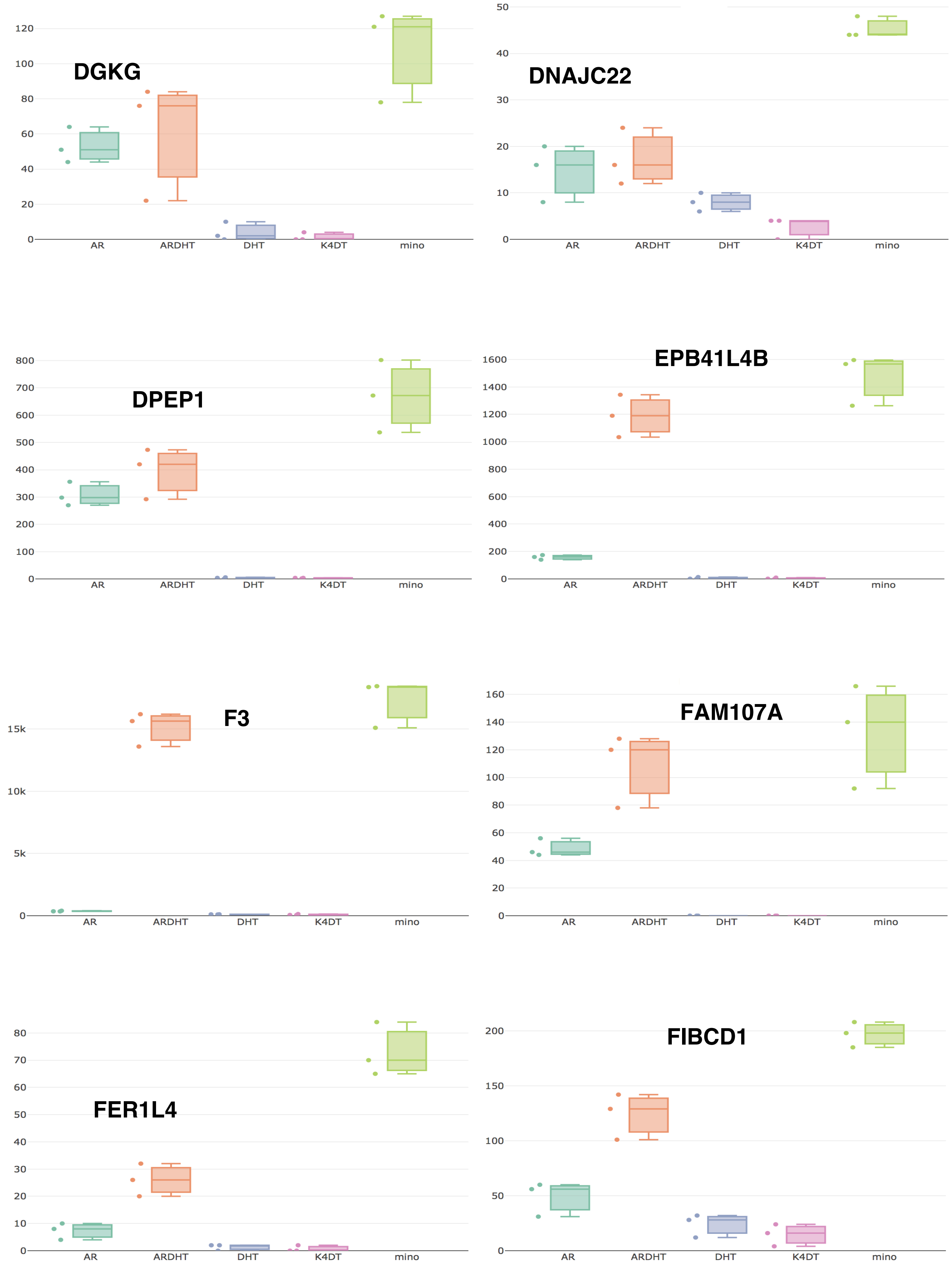
DGKG
AR ARDHT DHT K4DT mino
DNAJC22
AR ARDHT DHT K4DT mino
DPEP1
AR ARDHT DHT K4DT mino
EPB41L4B
AR ARDHT DHT K4DT mino
F3
AR ARDHT DHT K4DT mino
FAM107A
AR ARDHT DHT K4DT mino
FER1L4
AR ARDHT DHT K4DT mino
FIBCD1
AR ARDHT DHT K4DT mino

Figure S12. Bar plot mapping of 130 upregulated genes. Results of DGKG, DNAJC22, DPEP1, EPB41L4B, F3, FAM107A, FER1L4, FIBCD1 were shown. AR is HFDPC with AR expression and no ligand treatment. ARDHT is HFDPC with AR expression and ligand treated. DHT is HFDPC without AR expression and ligand treated. K4DT is HFDPC without AR expression and no ligand treatment. Mino is HFDPC with AR expression and ligand and Minoxidil sulfate (MXS) treated.

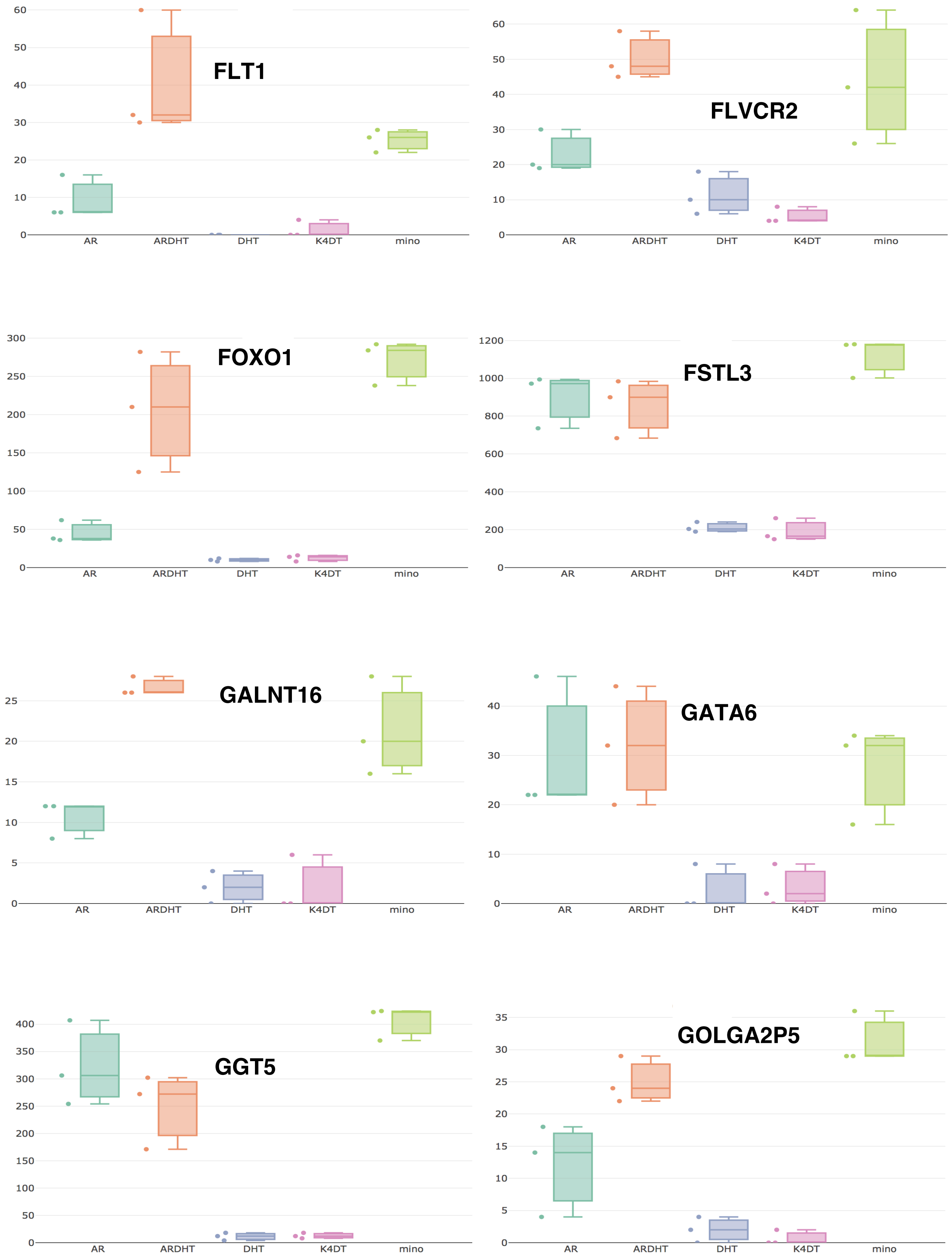
FLT1
FLVCR2
FOXO1
FSTL3
GALNT16
GATA6
GGT5
GOLGA2P5
AR
ARDHT
DHT
K4DT
mino

Figure S13. Bar plot mapping of 130 upregulated genes. Results of FLT1, FLVCR2, FOX1, FSTL3, GALNT16, GATA6, GGT5, GOLGA2P5 were shown. AR is HFDPC with AR expression and no ligand treatment. ARDHT is HFDPC with AR expression and ligand treated. DHT is HFDPC without AR expression and ligand treated. K4DT is HFDPC without AR expression and no ligand treatment. Mino is HFDPC with AR expression and ligand and Minoxidil sulfate (MXS) treated.

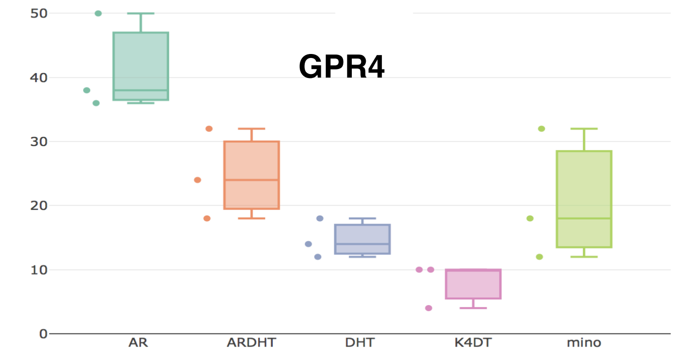
GPR4
50
40
30
20
10
0
AR
ARDHT
DHT
K4DT
mino

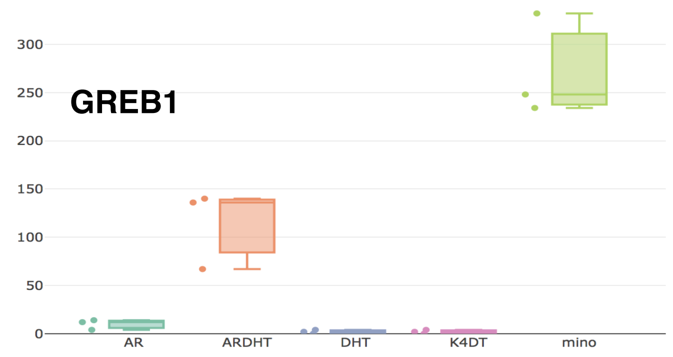
GREB1
300
250
200
150
100
50
0
AR
ARDHT
DHT
K4DT
mino

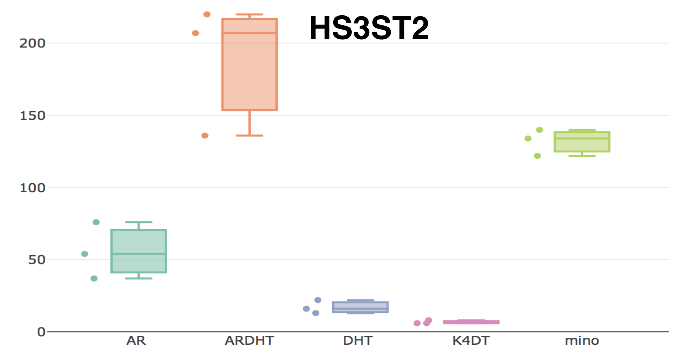
HS3ST2
200
150
100
50
0
AR
ARDHT
DHT
K4DT
mino

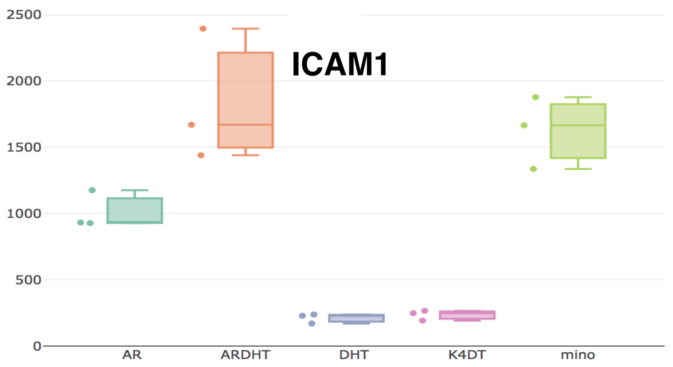
ICAM1
2500
2000
1500
1000
500
0
AR
ARDHT
DHT
K4DT
mino

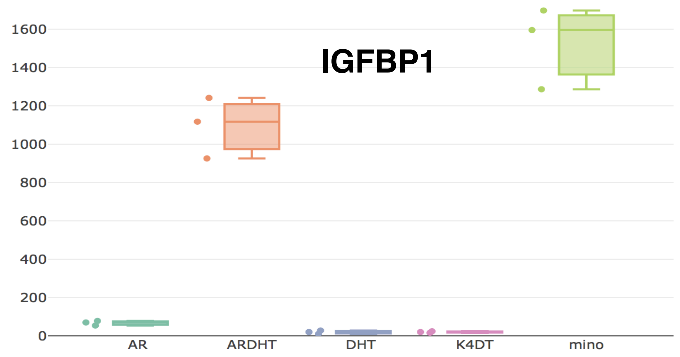
IGFBP1
1600
1400
1200
1000
800
600
400
200
0
AR
ARDHT
DHT
K4DT
mino

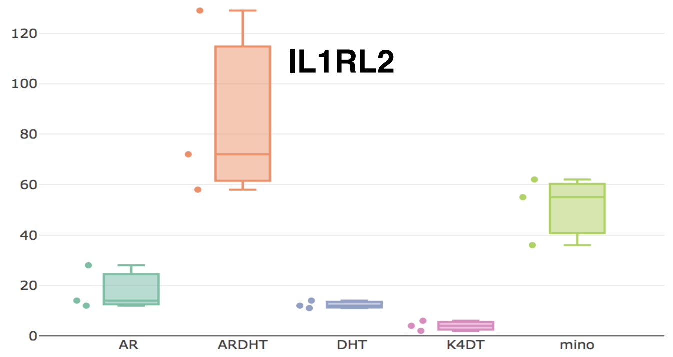
IL1RL2
120
100
80
60
40
20
0
AR
ARDHT
DHT
K4DT
mino

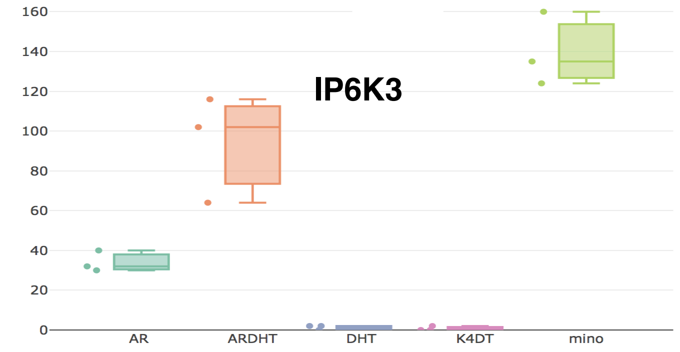
IP6K3
160
140
120
100
80
60
40
20
0
AR
ARDHT
DHT
K4DT
mino

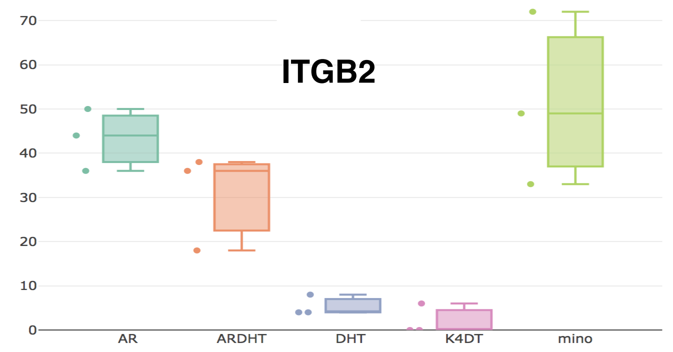
ITGB2
70
60
50
40
30
20
10
0
AR
ARDHT
DHT
K4DT
mino

Figure S14. Bar plot mapping of 130 upregulated genes. Results of GPR4, GREB1, HS3ST2, ICAM1, IGFBP1, ILRL2, IP6K3, ITGB2 were shown. AR is HFDPC with AR expression and no ligand treatment. ARDHT is HFDPC with AR expression and ligand treated. DHT is HFDPC without AR expression and ligand treated. K4DT is HFDPC without AR expression and no ligand treatment. Mino is HFDPC with AR expression and ligand and Minoxidil sulfate (MXS) treated.

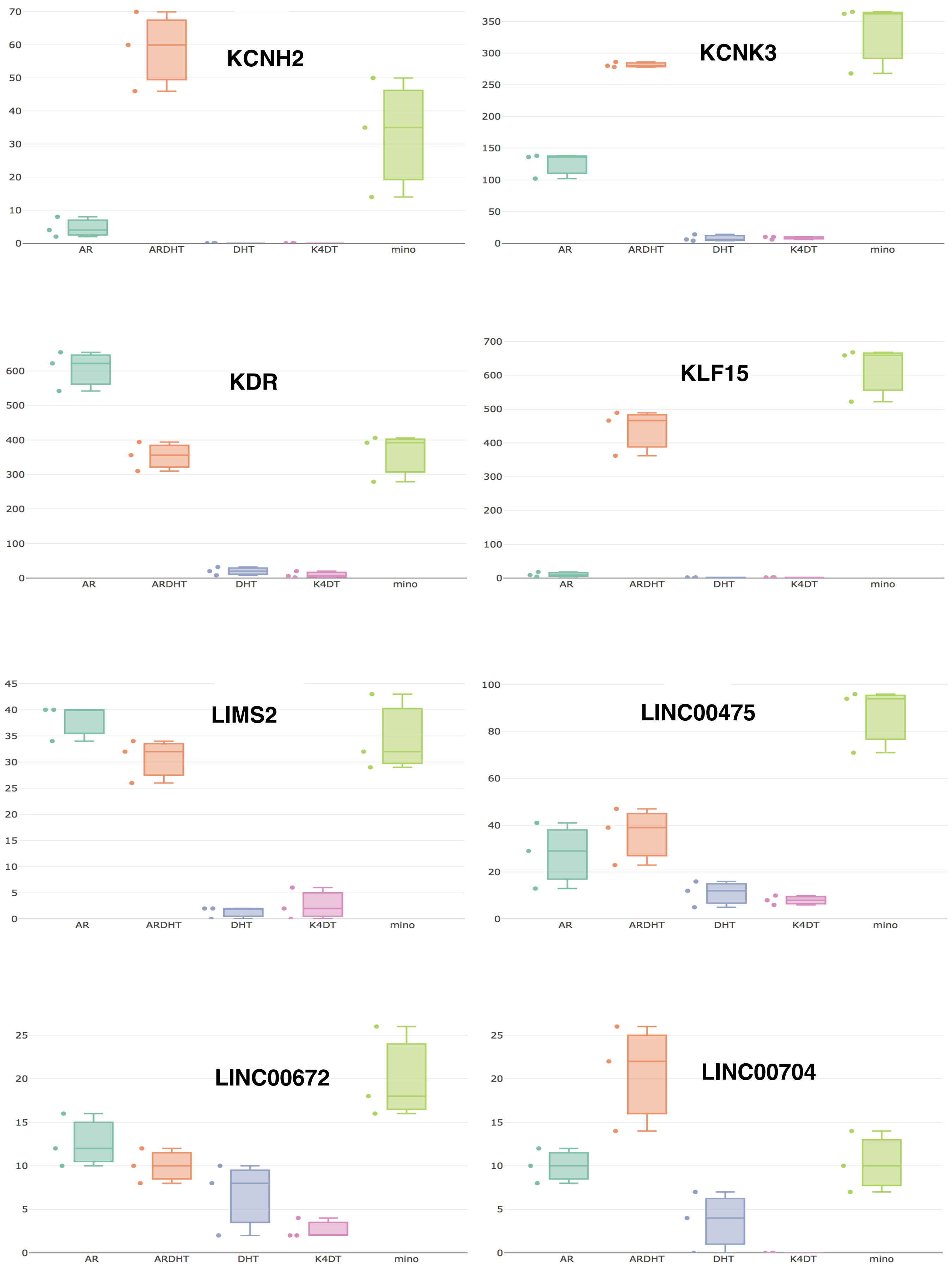
KCNH2
KCNK3
KDR
KLF15
LIMS2
LINC00475
LINC00672
LINC00704
AR
ARDHT
DHT
K4DT
mino

Figure S15. Bar plot mapping of 130 upregulated genes. Results of KCNH2, KCNK3, KDR, KLF15, LIMS2, LINC00475, LINC00672, LINC00704 were shown. AR is HFDPC with AR expression and no ligand treatment. ARDHT is HFDPC with AR expression and ligand treated. DHT is HFDPC without AR expression and ligand treated. K4DT is HFDPC without AR expression and no ligand treatment. Mino is HFDPC with AR expression and ligand and Minoxidil sulfate (MXS) treated.

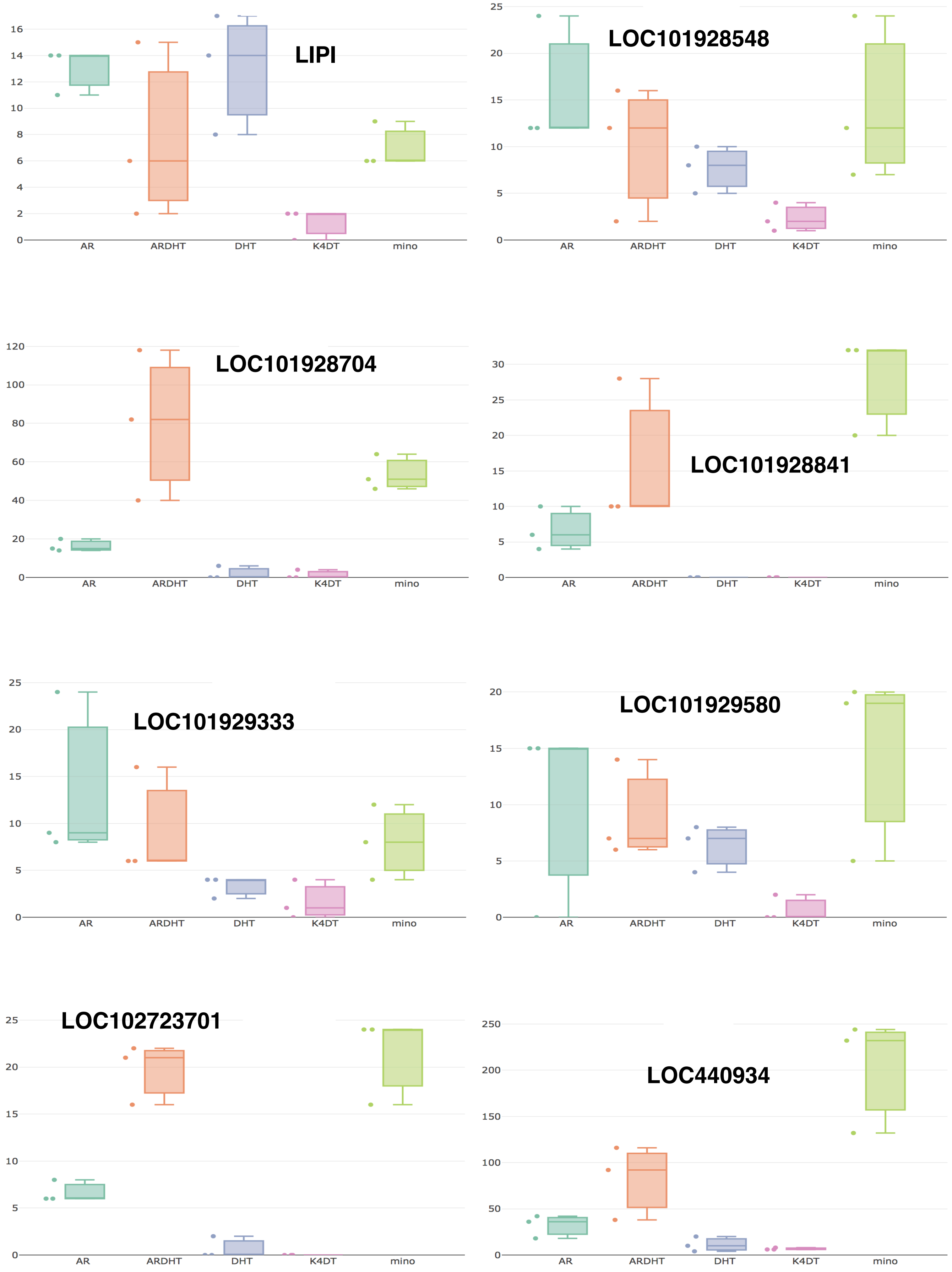
LIPI
LOC101928548
LOC101928704
LOC101928841
LOC101929333
LOC101929580
LOC102723701
LOC440934
AR
ARDHT
DHT
K4DT
mino

Figure S16. Bar plot mapping of 130 upregulated genes. Results of LIPI, LOC101928548, LOC101928704, LOC101928841, LOC101929333, LOC101929580, LOC102723701, LOC440934 were shown. AR is HFDPC with AR expression and no ligand treatment. ARDHT is HFDPC with AR expression and ligand treated. DHT is HFDPC without AR expression and ligand treated. K4DT is HFDPC without AR expression and no ligand treatment. Mino is HFDPC with AR expression and ligand and Minoxidil sulfate (MXS) treated.

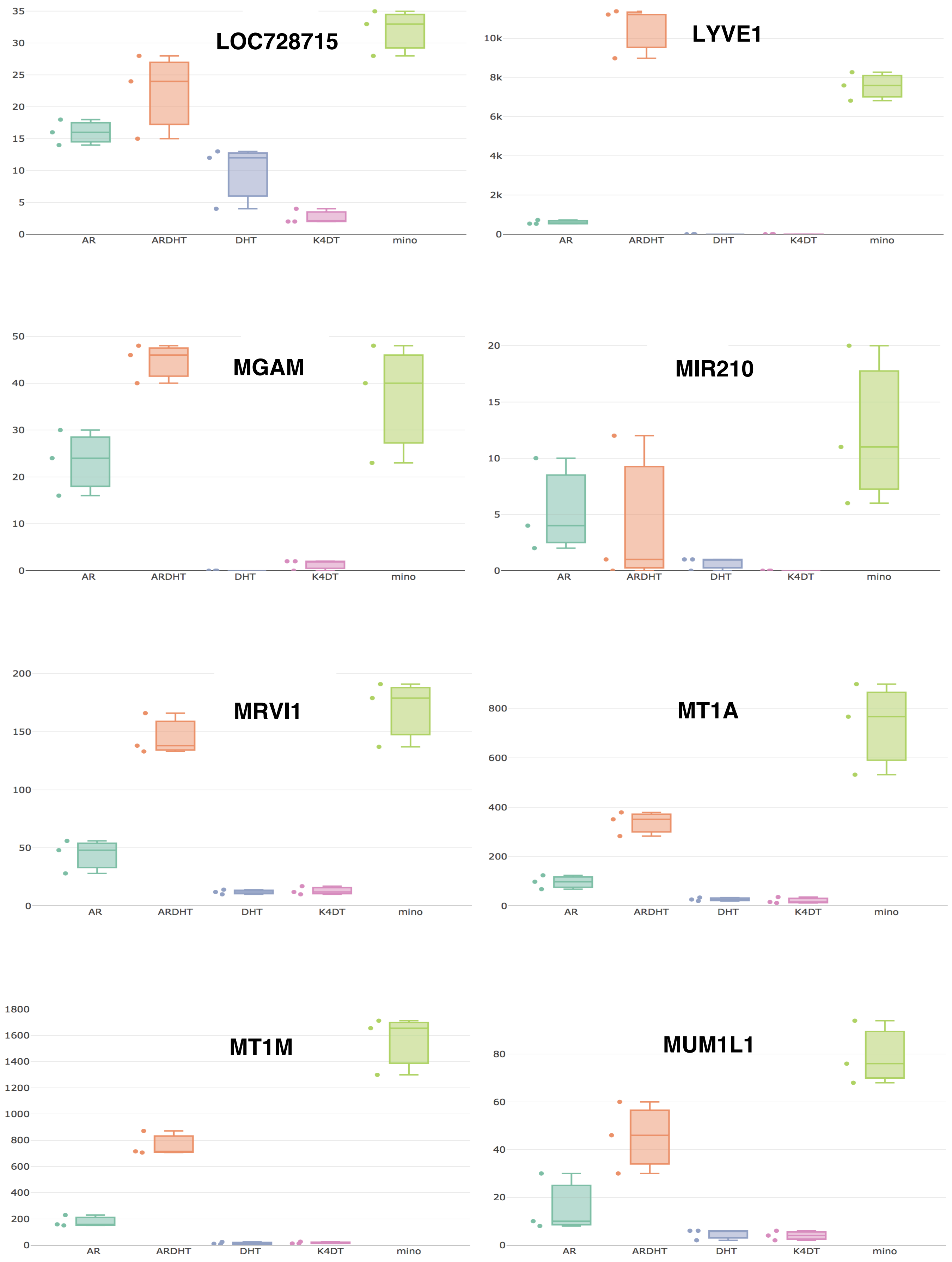

LOC728715
35
30
25
20
15
10
5
0
AR
ARDHT
DHT
K4DT
mino
LYVE1
10k
8k
6k
4k
2k
0
AR
ARDHT
DHT
K4DT
mino
MGAM
50
40
30
20
10
0
AR
ARDHT
DHT
K4DT
mino
MIR210
20
15
10
5
0
AR
ARDHT
DHT
K4DT
mino
MRVI1
200
150
100
50
0
AR
ARDHT
DHT
K4DT
mino
MT1A
800
600
400
200
0
AR
ARDHT
DHT
K4DT
mino
MT1M
1800
1600
1400
1200
1000
800
600
400
200
0
AR
ARDHT
DHT
K4DT
mino
MUM1L1
80
60
40
20
0
AR
ARDHT
DHT
K4DT
mino

Figure S17. Bar plot mapping of 130 upregulated genes. Results of LOC728715, LYVE1, MGAM, MIR210, MRVI1, MT1A, MT1M, MUM1L1 were shown. AR is HFDPC with AR expression and no ligand treatment. ARDHT is HFDPC with AR expression and ligand treated. DHT is HFDPC without AR expression and ligand treated. K4DT is HFDPC without AR expression and no ligand treatment. Mino is HFDPC with AR expression and ligand and Minoxidil sulfate (MXS) treated.

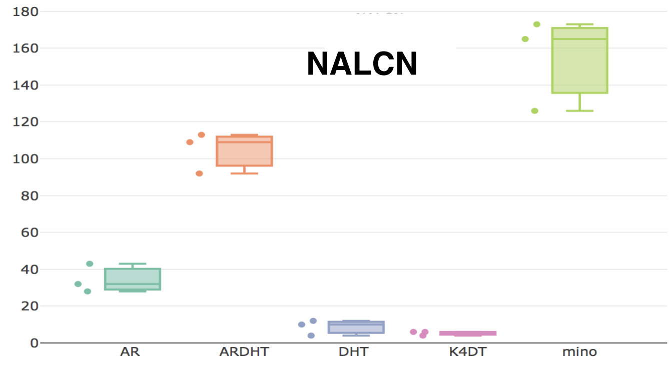
NALCN
180
160
140
120
100
80
60
40
20
0
AR
ARDHT
DHT
K4DT
mino

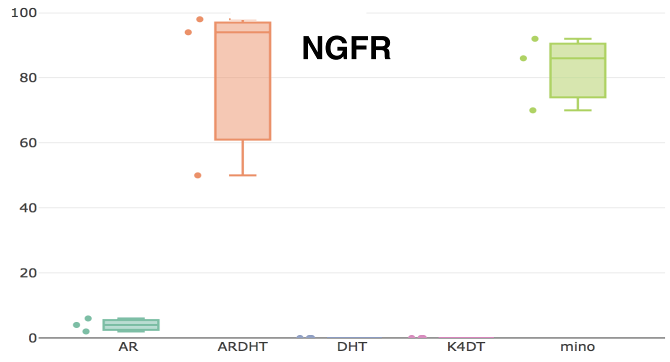
NGFR
100
80
60
40
20
0
AR
ARDHT
DHT
K4DT
mino

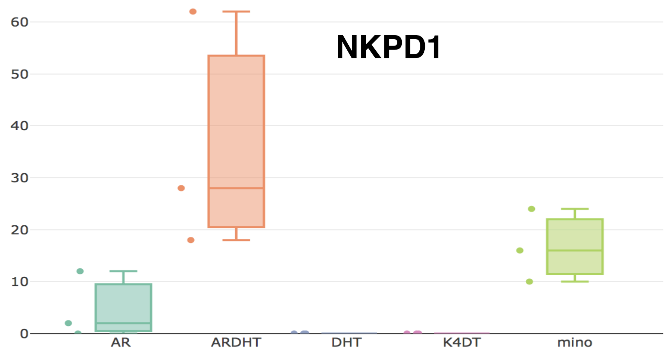
NKPD1
60
50
40
30
20
10
0
AR
ARDHT
DHT
K4DT
mino

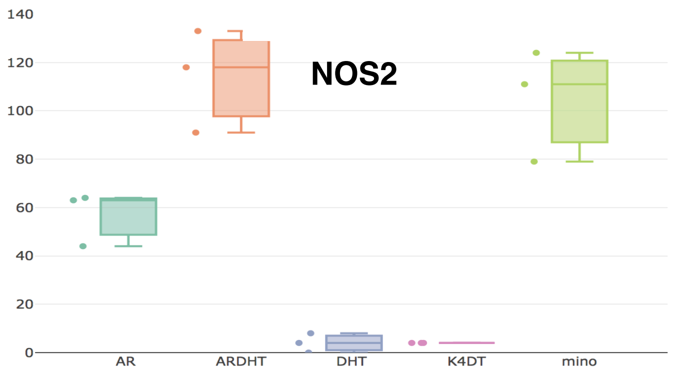
NOS2
140
120
100
80
60
40
20
0
AR
ARDHT
DHT
K4DT
mino

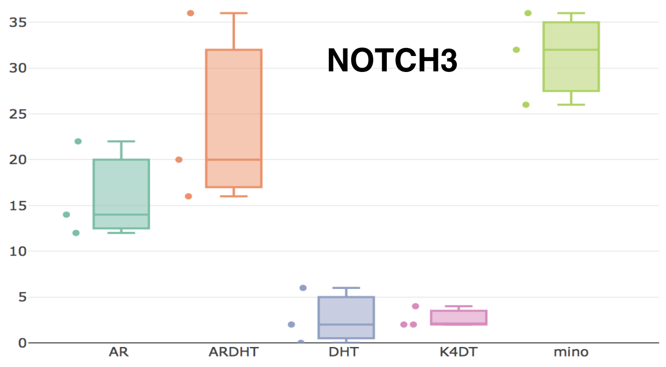
NOTCH3
35
30
25
20
15
10
5
0
AR
ARDHT
DHT
K4DT
mino

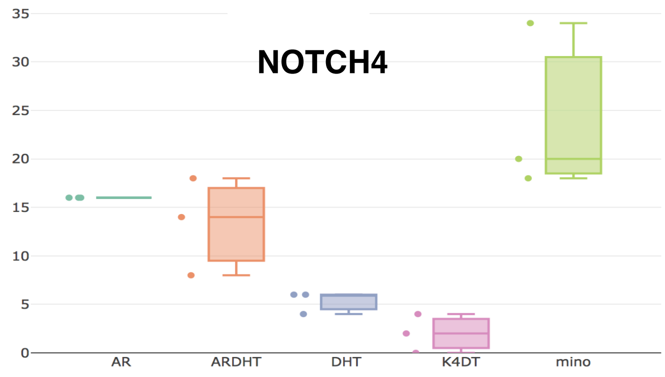
NOTCH4
35
30
25
20
15
10
5
0
AR
ARDHT
DHT
K4DT
mino

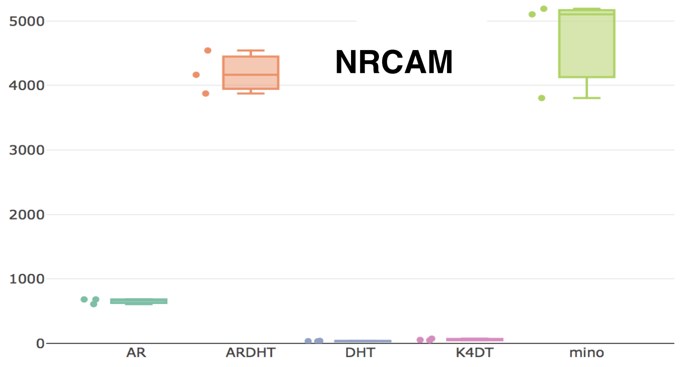
NRCAM
5000
4000
3000
2000
1000
0
AR
ARDHT
DHT
K4DT
mino

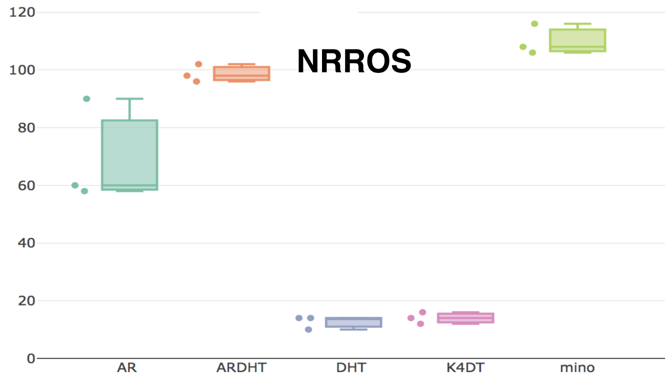
NRROS
120
100
80
60
40
20
0
AR
ARDHT
DHT
K4DT
mino

Figure S18. Bar plot mapping of 130 upregulated genes. Results of NALCN, NGFR, NKPD1, NOS2, NOTCH3, NOTCH4, NRCAM, NRROS were shown. AR is HFDPC with AR expression and no ligand treatment. ARDHT is HFDPC with AR expression and ligand treated. DHT is HFDPC without AR expression and ligand treated. K4DT is HFDPC without AR expression and no ligand treatment. Mino is HFDPC with AR expression and ligand and Minoxidil sulfate (MXS) treated.

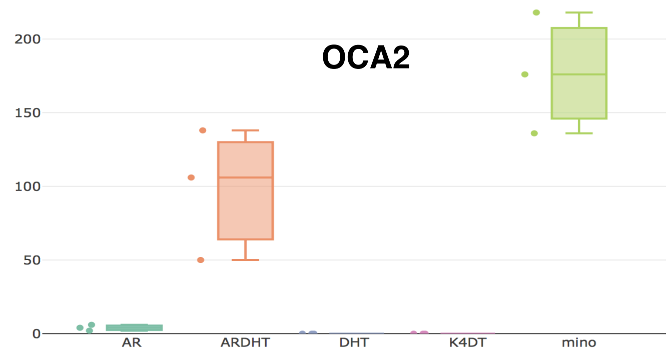
OCA2
200
150
100
50
0
AR
ARDHT
DHT
K4DT
mino

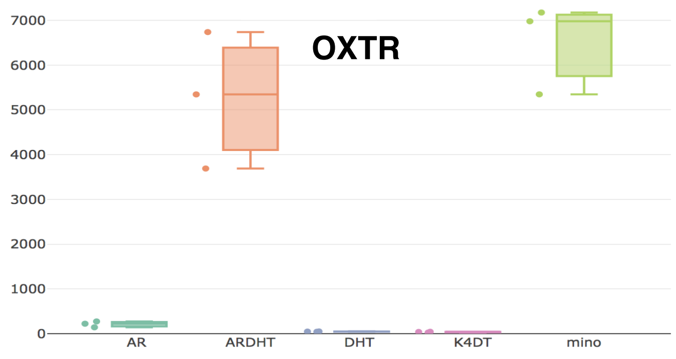
OXTR
7000
6000
5000
4000
3000
2000
1000
0
AR
ARDHT
DHT
K4DT
mino

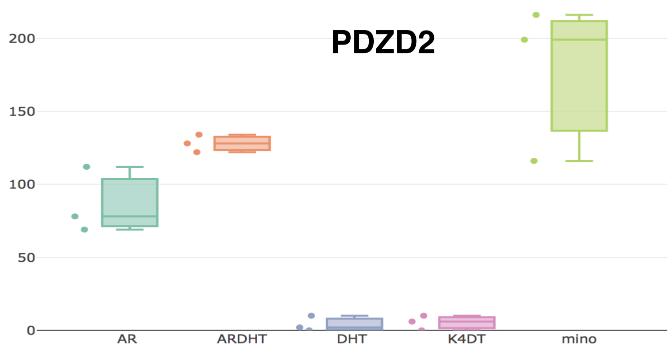
PDZD2
200
150
100
50
0
AR
ARDHT
DHT
K4DT
mino

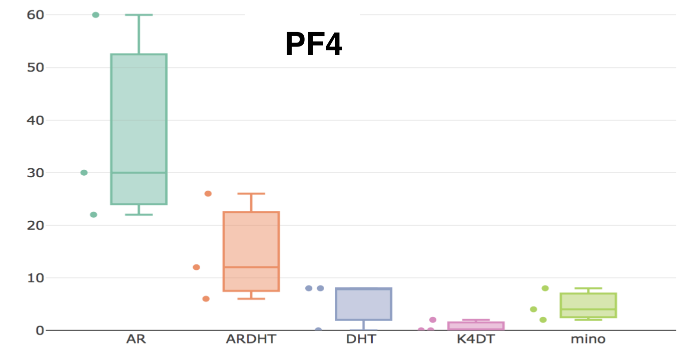
PF4
60
50
40
30
20
10
0
AR
ARDHT
DHT
K4DT
mino

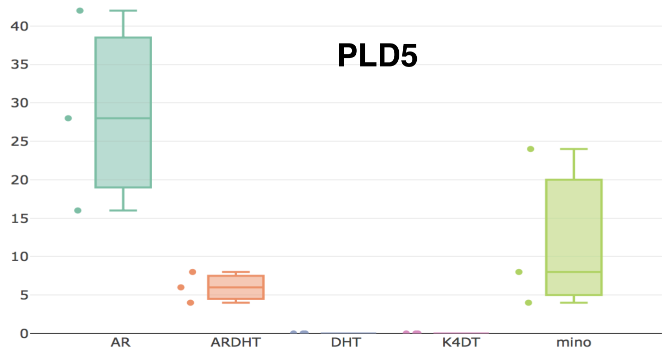
PLD5
40
35
30
25
20
15
10
5
0
AR
ARDHT
DHT
K4DT
mino

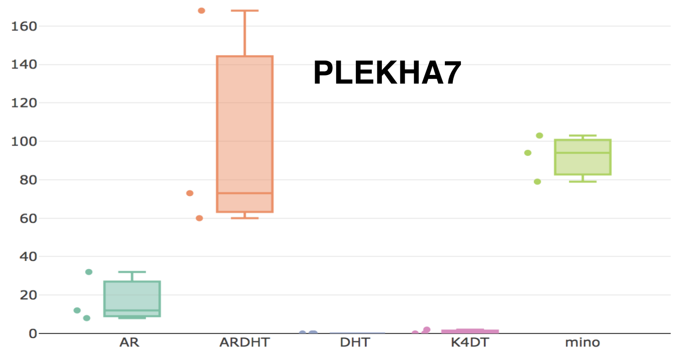
PLEKHA7
160
140
120
100
80
60
40
20
0
AR
ARDHT
DHT
K4DT
mino

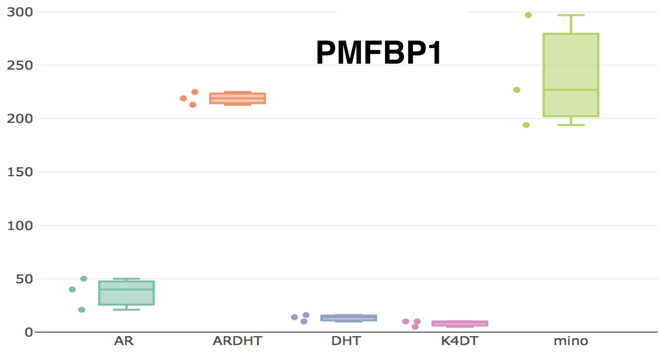
PMFBP1
300
250
200
150
100
50
0
AR
ARDHT
DHT
K4DT
mino

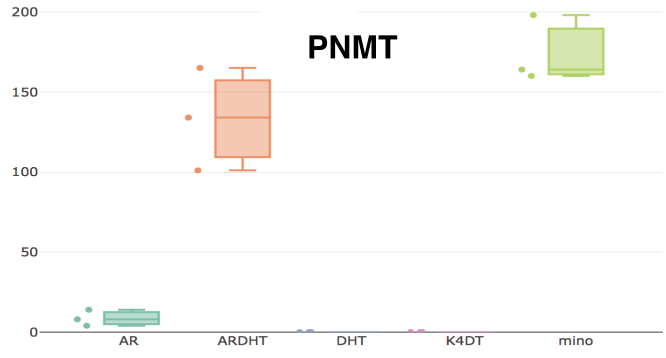
PNMT
200
150
100
50
0
AR
ARDHT
DHT
K4DT
mino

Figure S19. Bar plot mapping of 130 upregulated genes. Results of OCA2, OXTR, PDZD2, PF4, PLD5, PLEKHA7, PMFBP1, PNMT were shown. AR is HFDPC with AR expression and no ligand treatment. ARDHT is HFDPC with AR expression and ligand treated. DHT is HFDPC without AR expression and ligand treated. K4DT is HFDPC without AR expression and no ligand treatment. Mino is HFDPC with AR expression and ligand and Minoxidil sulfate (MXS) treated.

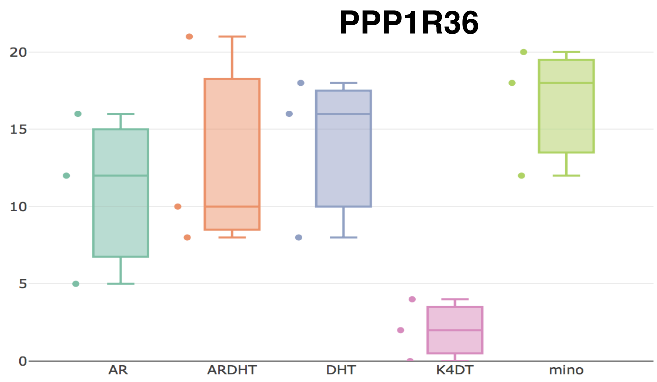
PPP1R36
20
15
10
5
0
AR
ARDHT
DHT
K4DT
mino

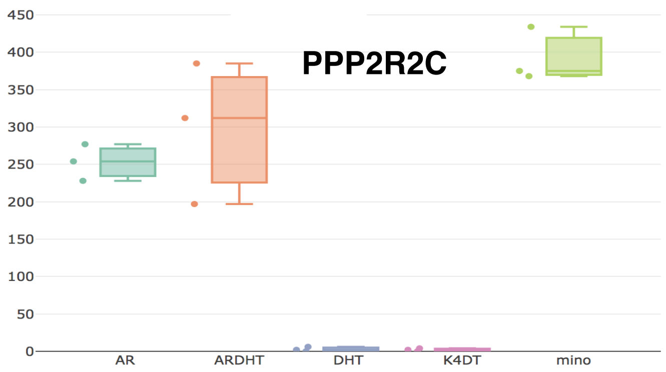
PPP2R2C
450
400
350
300
250
200
150
100
50
0
AR
ARDHT
DHT
K4DT
mino

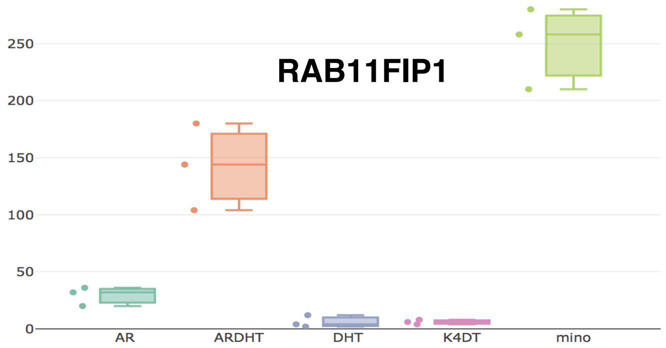
RAB11FIP1
250
200
150
100
50
0
AR
ARDHT
DHT
K4DT
mino

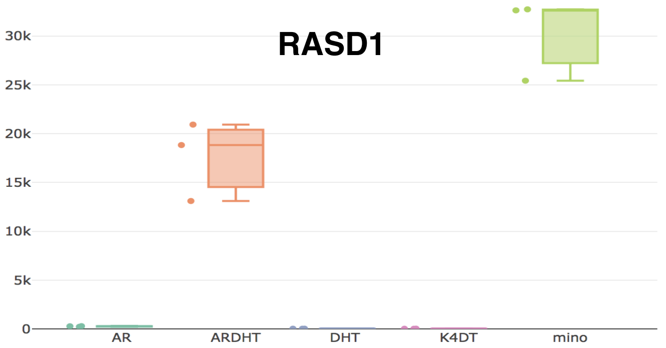
RASD1
30k
25k
20k
15k
10k
5k
0
AR
ARDHT
DHT
K4DT
mino

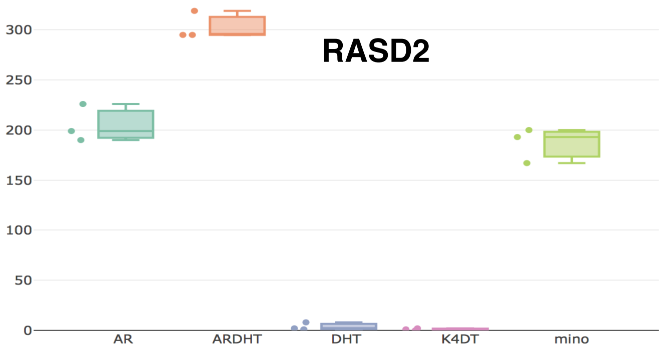
RASD2
300
250
200
150
100
50
0
AR
ARDHT
DHT
K4DT
mino

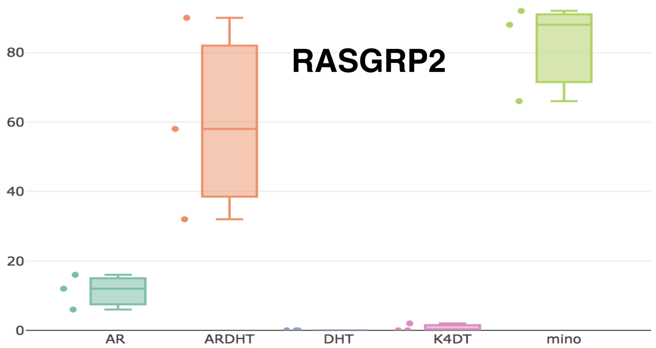
RASGRP2
80
60
40
20
0
AR
ARDHT
DHT
K4DT
mino

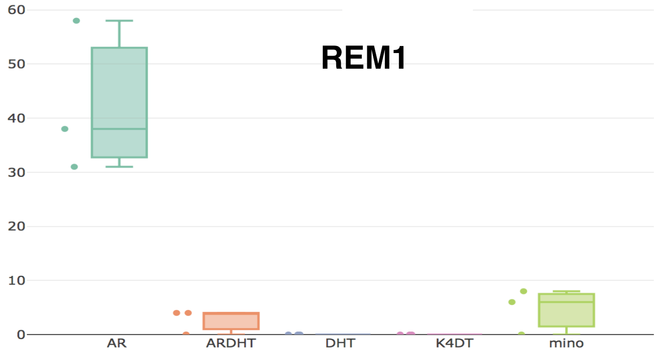
REM1
60
50
40
30
20
10
0
AR
ARDHT
DHT
K4DT
mino

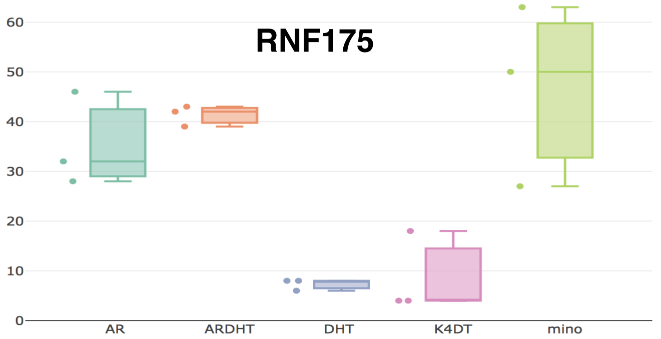
RNF175
60
50
40
30
20
10
0
AR
ARDHT
DHT
K4DT
mino

Figure S20. Bar plot mapping of 130 upregulated genes. Results of RAB11FIP1, RASD1, RASD2, RASGRP2, REM1, RNF175 were shown. AR is HFDPC with AR expression and no ligand treatment. ARDHT is HFDPC with AR expression and ligand treated. DHT is HFDPC without AR expression and ligand treated. K4DT is HFDPC without AR expression and no ligand treatment. Mino is HFDPC with AR expression and ligand and Minoxidil sulfate (MXS) treated.

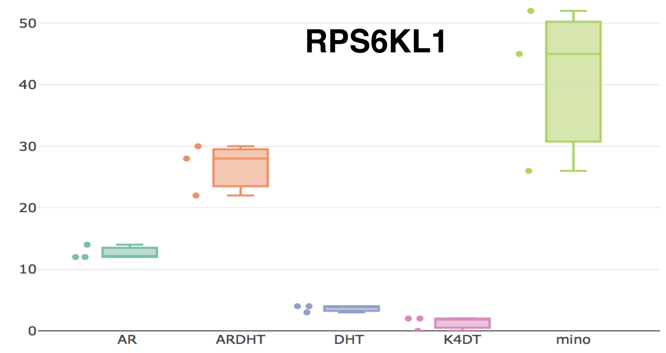
RPS6KL1
50
40
30
20
10
0
AR
ARDHT
DHT
K4DT
mino

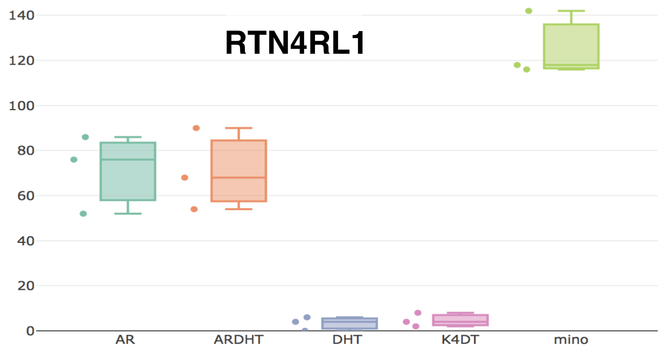
RTN4RL1
140
120
100
80
60
40
20
0
AR
ARDHT
DHT
K4DT
mino

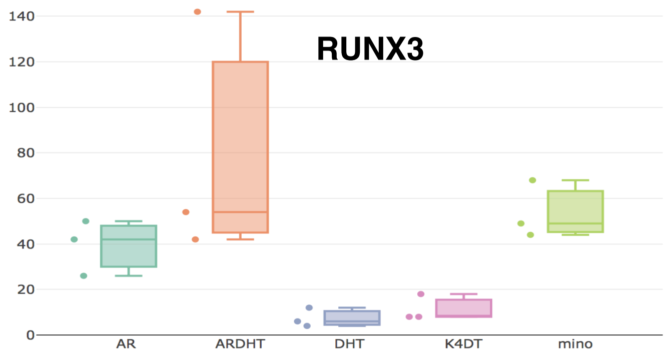
RUNX3
140
120
100
80
60
40
20
0
AR
ARDHT
DHT
K4DT
mino

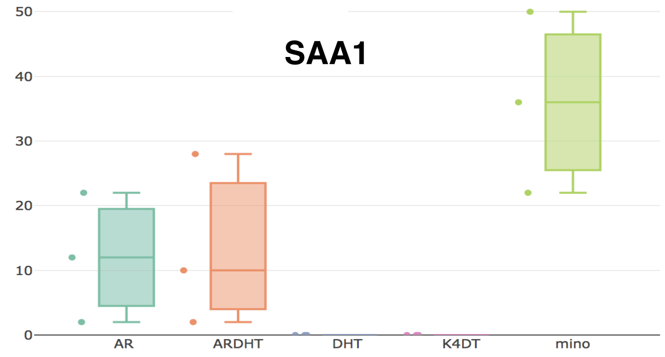
SAA1
50
40
30
20
10
0
AR
ARDHT
DHT
K4DT
mino

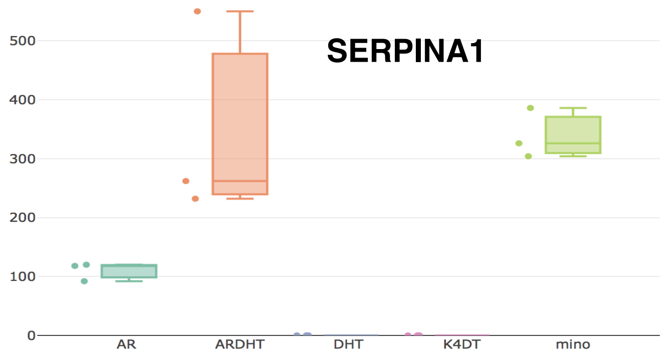
SERPINA1
500
400
300
200
100
0
AR
ARDHT
DHT
K4DT
mino

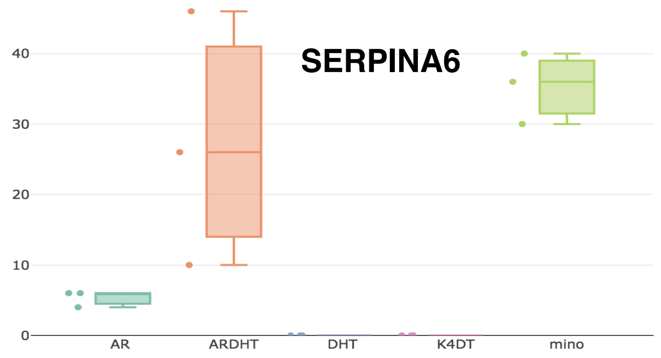
SERPINA6
40
30
20
10
0
AR
ARDHT
DHT
K4DT
mino

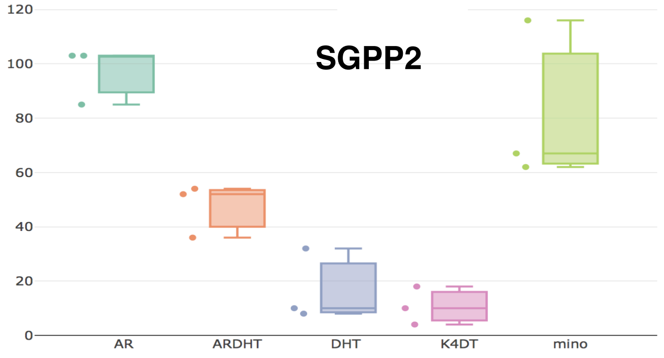
SGPP2
120
100
80
60
40
20
0
AR
ARDHT
DHT
K4DT
mino

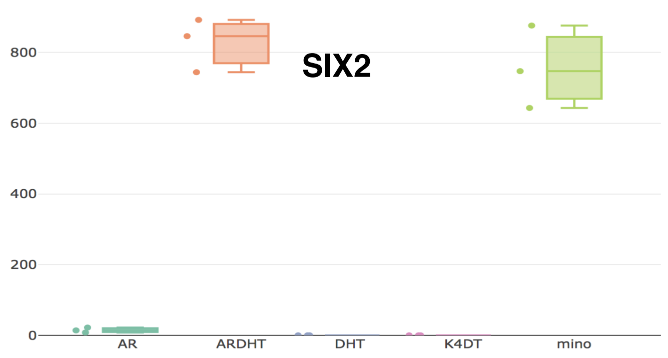
SIX2
800
600
400
200
0
AR
ARDHT
DHT
K4DT
mino

Figure S21. Bar plot mapping of 130 upregulated genes. Results of RPS6KL1, RTN4RL1, RUNX3, SAA1, SERPINA1, SERPINA6, SGPP2, SIX2 were shown. AR is HFDPC with AR expression and no ligand treatment. ARDHT is HFDPC with AR expression and ligand treated. DHT is HFDPC without AR expression and ligand treated. K4DT is HFDPC without AR expression and no ligand treatment. Mino is HFDPC with AR expression and ligand and Minoxidil sulfate (MXS) treated.

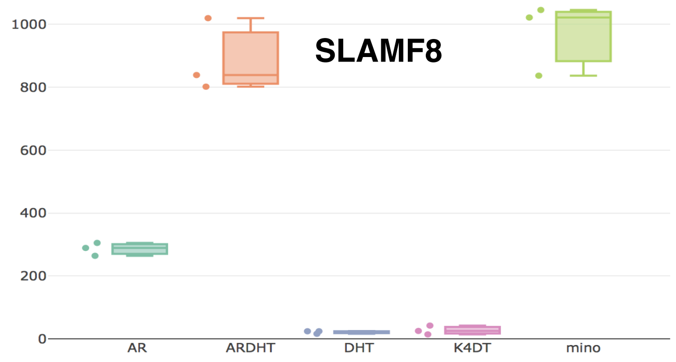
SLAMF8
1000
800
600
400
200
0
AR
ARDHT
DHT
K4DT
mino

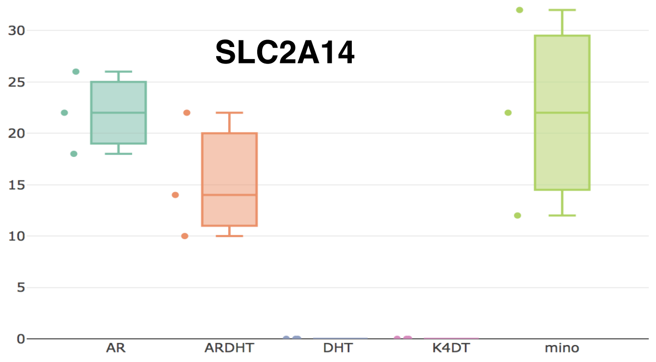
SLC2A14
30
25
20
15
10
5
0
AR
ARDHT
DHT
K4DT
mino

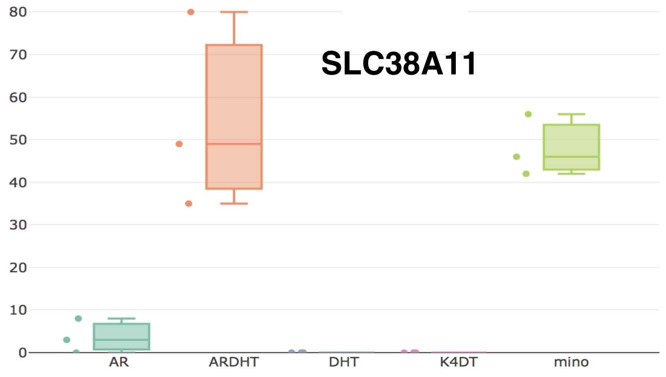
SLC38A11
80
70
60
50
40
30
20
10
0
AR
ARDHT
DHT
K4DT
mino

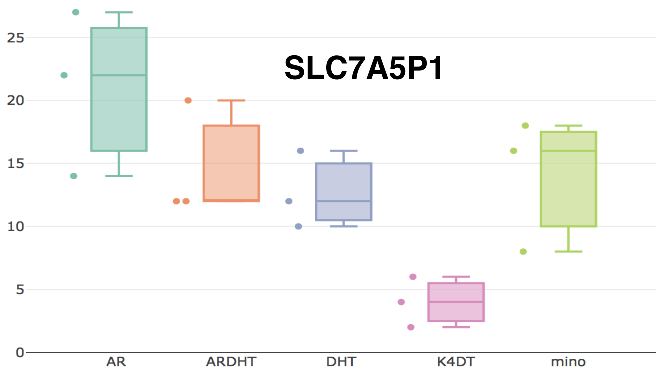
SLC7A5P1
25
20
15
10
5
0
AR
ARDHT
DHT
K4DT
mino

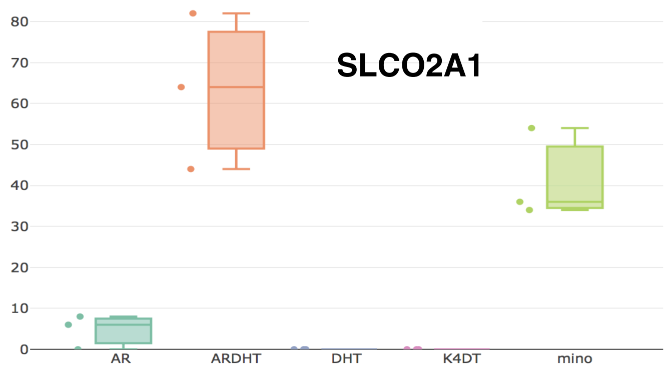
SLCO2A1
80
70
60
50
40
30
20
10
0
AR
ARDHT
DHT
K4DT
mino

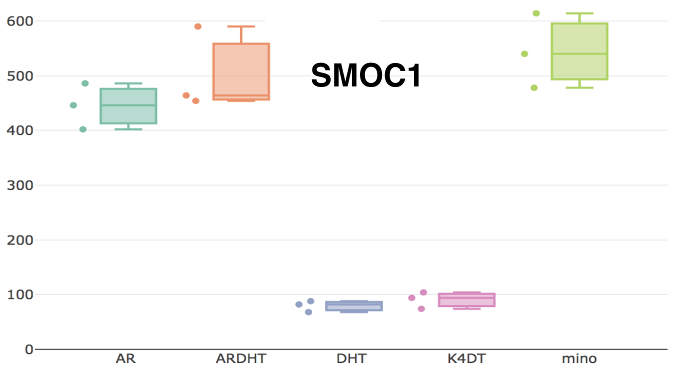
SMOC1
600
500
400
300
200
100
0
AR
ARDHT
DHT
K4DT
mino

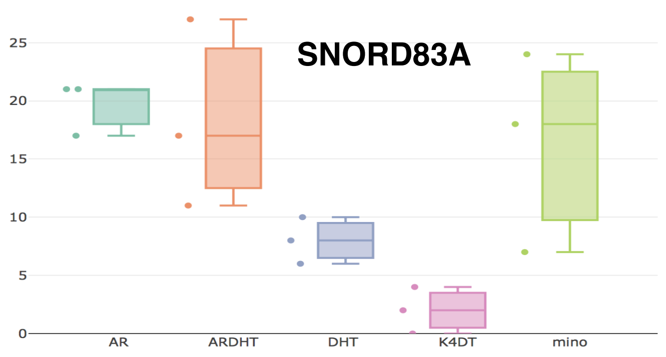
SNORD83A
25
20
15
10
5
0
AR
ARDHT
DHT
K4DT
mino

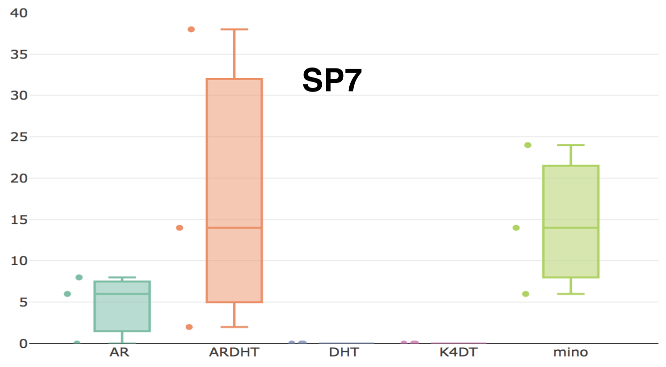
SP7
40
35
30
25
20
15
10
5
0
AR
ARDHT
DHT
K4DT
mino

Figure S22. Bar plot mapping of 130 upregulated genes. Results of SLAMF8, SLC2A14, SLC38A11, SLC7A5P1, SLCO2A1, SMOC1, SNORD83A, SP7 were shown. AR is HFDPC with AR expression and no ligand treatment. ARDHT is HFDPC with AR expression and ligand treated. DHT is HFDPC without AR expression and ligand treated. K4DT is HFDPC without AR expression and no ligand treatment. Mino is HFDPC with AR expression and ligand and Minoxidil sulfate (MXS) treated.

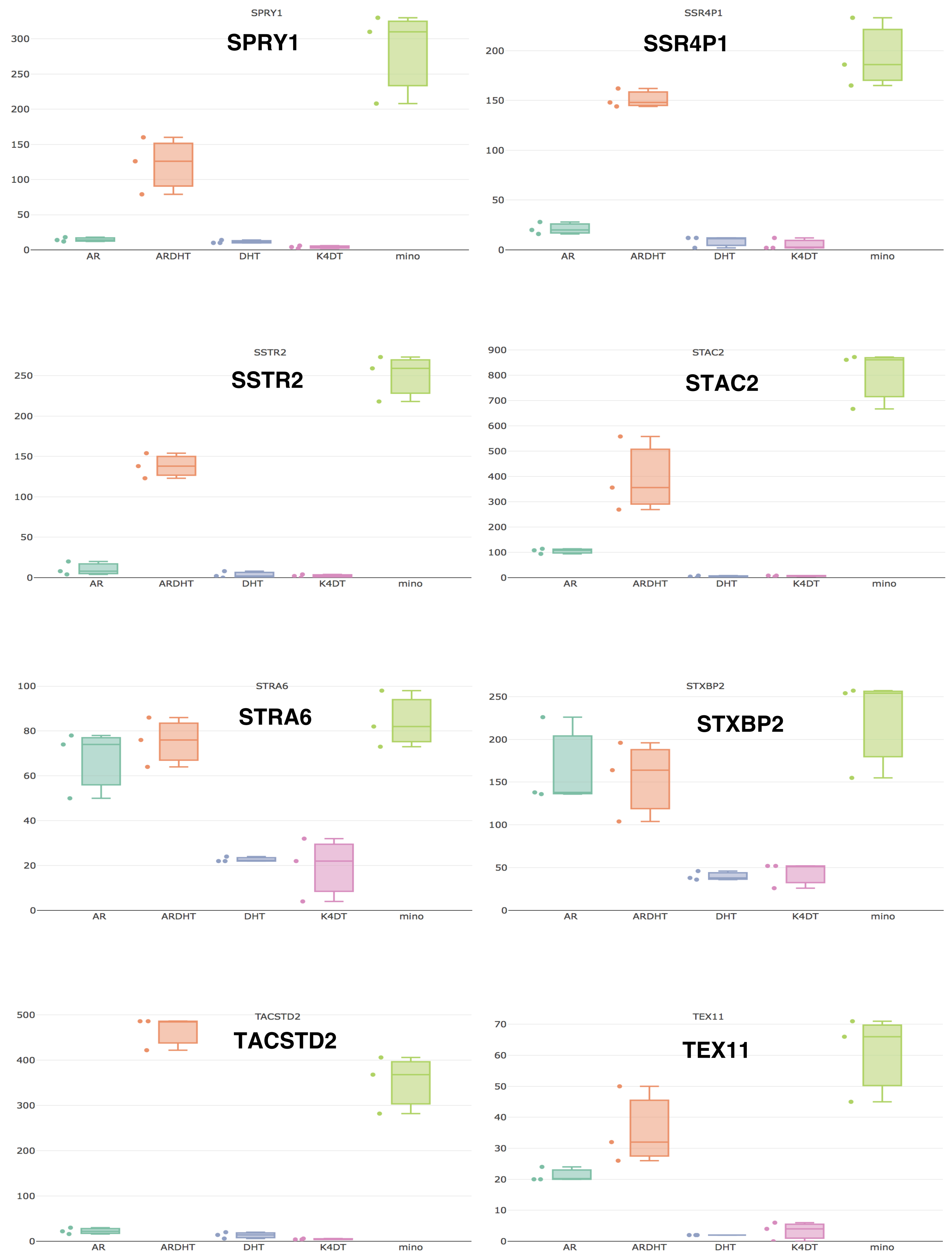

SPRY1
SPRY1
0
50
100
150
200
250
300
AR
ARDHT
DHT
K4DT
mino
SSR4P1
SSR4P1
0
50
100
150
200
AR
ARDHT
DHT
K4DT
mino
SSTR2
SSTR2
0
50
100
150
200
250
AR
ARDHT
DHT
K4DT
mino
STAC2
STAC2
0
100
200
300
400
500
600
700
800
900
AR
ARDHT
DHT
K4DT
mino
STRA6
STRA6
0
20
40
60
80
100
AR
ARDHT
DHT
K4DT
mino
STXBP2
STXBP2
0
50
100
150
200
250
AR
ARDHT
DHT
K4DT
mino
TACSTD2
TACSTD2
0
100
200
300
400
500
AR
ARDHT
DHT
K4DT
mino
TEX11
TEX11
0
10
20
30
40
50
60
70
AR
ARDHT
DHT
K4DT
mino

Figure S23. Bar plot mapping of 130 upregulated genes. Results of SPRY1, SSR4P1, SSTR2, STAC2, STRA6, STXBP2, TACSTD2, TEX11 were shown. AR is HFDPC with AR expression and no ligand treatment. ARDHT is HFDPC with AR expression and ligand treated. DHT is HFDPC without AR expression and ligand treated. K4DT is HFDPC without AR expression and no ligand treatment. Mino is HFDPC with AR expression and ligand and Minoxidil sulfate (MXS) treated.

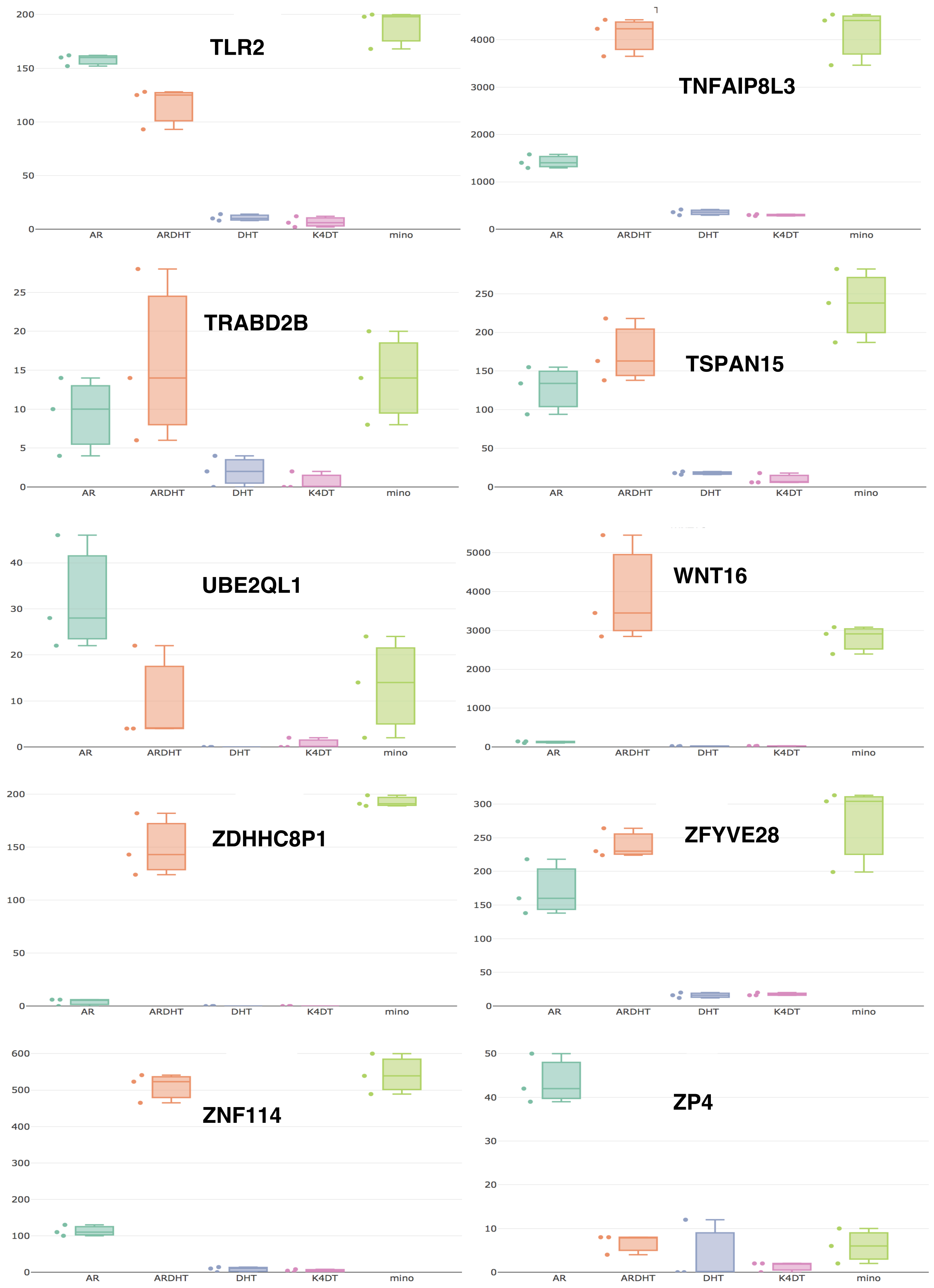
TLR2
200
150
100
50
0
AR
ARDHT
DHT
K4DT
mino
TNFAIP8L3
4000
3000
2000
1000
0
AR
ARDHT
DHT
K4DT
mino
TRABD2B
25
20
15
10
5
0
AR
ARDHT
DHT
K4DT
mino
TSPAN15
250
200
150
100
50
0
AR
ARDHT
DHT
K4DT
mino
UBE2QL1
40
30
20
10
0
AR
ARDHT
DHT
K4DT
mino
WNT16
5000
4000
3000
2000
1000
0
AR
ARDHT
DHT
K4DT
mino
ZDHHC8P1
200
150
100
50
0
AR
ARDHT
DHT
K4DT
mino
ZFYVE28
300
250
200
150
100
50
0
AR
ARDHT
DHT
K4DT
mino
ZNF114
600
500
400
300
200
100
0
AR
ARDHT
DHT
K4DT
mino
ZP4
50
40
30
20
10
0
AR
ARDHT
DHT
K4DT
mino

Figure S24. Bar plot mapping of 130 upregulated genes. Results of TLR2, TNFAIP8L3, TRABD2B, TSPAN15, UBE2QL1, WNT16, ZDHHC8P1, ZFYVE28, ZNF114, ZP4 were shown. AR is HFDPC with AR expression and no ligand treatment. ARDHT is HFDPC with AR expression and ligand treated. DHT is HFDPC without AR expression and ligand treated. K4DT is HFDPC without AR expression and no ligand treatment. Mino is HFDPC with AR expression and ligand and Minoxidil sulfate (MXS) treated.
